# Bioprinting-Enabled Customization of Biohybrid Actuators: Scaling Dimensions and Functional Complexity

**DOI:** 10.3390/gels12100923

**Published:** 2026-10-14

**Authors:** Maede Pajouhande, Nehar Celikkin, Saeed Rafieyan, Maria Celeste Tirelli, Fabio Maiullari, Maryia Piletskaya, Marco Costantini

**Affiliations:** Institute of Physical Chemistry, Polish Academy of Sciences, Kasprzaka 44/52, 01-224 Warsaw, Poland; mpajouhande@ichf.edu.pl (M.P.); ncelikkn@ichf.edu.pl (N.C.); saeed.rafieyan@ichf.edu.pl (S.R.); mctirelli@ichf.edu.pl (M.C.T.); fmaiullari@ichf.edu.pl (F.M.); mpiletskaya@ichf.edu.pl (M.P.)

**Keywords:** biohybrid robotics, muscle-based actuators, soft robotics, 3D bioprinting, biofabrication, muscle tissue engineering

## Abstract

Biohybrid actuators integrate living contractile cells with engineered materials, combining biological force generation with the structural and functional control offered by synthetic components. However, translating engineered muscle tissue into reproducible, high-performance biohybrid robots remains limited by tissue organization, matrix degradation, force transmission, and long-term functional stability. This review analyzes how biofabrication parameters and material properties influence tissue maturation and actuator-level performance, with particular emphasis on bioprinting. We compare muscle cell sources; extrusion-based, embedded, and light-assisted fabrication strategies; and scaffold biomaterials using reported structural and functional outcomes. We also evaluate electrical and optical activation and mechanical conditioning, distinguishing their roles in controlling contraction and promoting maturation. The comparison highlights a gap between architectural control during fabrication and reproducible robotic performance: methods that produce complex muscle constructs do not necessarily yield integrated devices with validated force transmission or locomotion. Tissue maturation, tissue–material interfaces, and nutrient transport emerge as closely coupled priorities for scaling functional constructs. We conclude with a framework for standardized benchmarking and discuss multimaterial printing, data-driven design, and feedback control as complementary routes toward more reproducible and capable biohybrid actuators.

## 1. Introduction

Engineers have long drawn inspiration from nature to address technological challenges. An insect-scale aerial robot flown under closed-loop control by soft artificial muscles [1], design principles for energy and control autonomy abstracted from living systems [2], and a lizard-inspired robot used to identify how limb and spine coordination trades off speed, efficiency and stability [3] together illustrate how biological organization serves as a blueprint for engineered devices. Yet, purely synthetic, bio-inspired devices still face challenges in adaptability and self-repair compared to living systems.

Biohybrid robots integrate at least one biological component for sensing, information processing, or actuation [4]. While advances in biomimetic sensing and control have significantly progressed the perception and decision-making capabilities of biohybrid robotics, muscle-based actuation offers a complementary route to biologically integrated movement. Accordingly, living biohybrid actuators are increasingly recognized as a promising component of biohybrid robotics, owing to their potential to provide adaptive force generation, intrinsic compliance, and biologically inspired interactions that remain difficult to achieve with fully synthetic systems.

Over the past 15 years, swimming, rotating, walking, and gripping biohybrid actuators have been proposed for a wide range of applications, including drug delivery, fluid actuation, bio-imaging, and cardiac tissue repair [5]. The diverse motion capabilities of biohybrid actuators have been engineered using various contracting cell types, biomaterials, and biofabrication techniques. Early biohybrid actuators fabricated using manual assembly or molding demonstrated functional motion but also highlighted the challenges associated with scaling up, including diffusion-limited thickness, repeatability issues, and operator dependency [6]. Accordingly, automated fabrication strategies, including bioprinting, have attracted significant attention for addressing these challenges and improving reproducibility, scalability, and automation. The term “scaling” as used throughout this review refers to the physical and structural challenge of increasing construct dimensions, ranging from thin planar films to volumetric three-dimensional tissues, while preserving cellular organization, viability, and contractile function. Bioprinting is central to this challenge because it enables systematic, reproducible deposition of cells and matrix at defined spatial coordinates, making it possible to build larger constructs with controlled architecture that would be difficult to achieve through manual or mold-based methods. Scaling construct dimensions in this way is a prerequisite for increasing force output and integrating muscle tissues into larger robotic platforms. Three forms of scalability should be distinguished. Geometric scalability refers to the dimensions a fabrication method can produce, biological scalability to the dimensions over which cells remain viable and contractile, and manufacturing scalability to production throughput and reproducibility. These forms of scalability do not necessarily advance together; biological scalability depends on transport and maturation, as well as fabrication capability. The following calculation is an illustrative transport model rather than a measured viability threshold, but it makes the coupling explicit. For a long cylindrical construct of radius *r*, neglecting its ends, the lateral surface-area-to-volume ratio is 2/r, so a tenfold increase in diameter reduces the exchange surface available per unit volume by the same factor. If active stress, alignment, and recruitment remain constant, force scales with the active cross-sectional area and hence with $r^{2}$. The normalization matters here and is often left unstated: specific force may be reported against the total construct cross-section, against the viable or contractile fraction of that cross-section, or against a physiological cross-sectional area corrected for fibre pennation, and these are not interchangeable [7,8]. Diffusion-supported tissue thickness is commonly estimated at 100–200 µm from a supply source, although the limit depends on cell density, metabolic demand, and transport conditions [9]. Taking a representative penetration depth d=150 µm, the supplied fraction of a cylindrical cross-section is $1-{\left(1-d/R\right)}^{2}$. A construct of 0.3 mm diameter (R=150 µm) then lies entirely within diffusion range, whereas at 3 mm diameter (R=1.5 mm) the supplied fraction is $1-{\left(1-0.15/1.5\right)}^{2}=0.19$, or roughly one fifth of the cross-section. The two scalings then work against each other: if only the outer shell is metabolically supported, the active area is the annulus $\pi [R^{2}-{\left(R-d\right)}^{2}]\approx 2\pi Rd$ for d≪R, so force grows in proportion to *R* rather than $R^{2}$. In this simplified model, increasing diameter without improving transport yields a hypoxic core and diminishing returns in force rather than a proportional gain. Perfusable channels or engineered vasculature could relieve the transport limit [10,11], but active stress and force transmission would have to be maintained as well for the potential gain in force to be realized.

**Figure 1:**
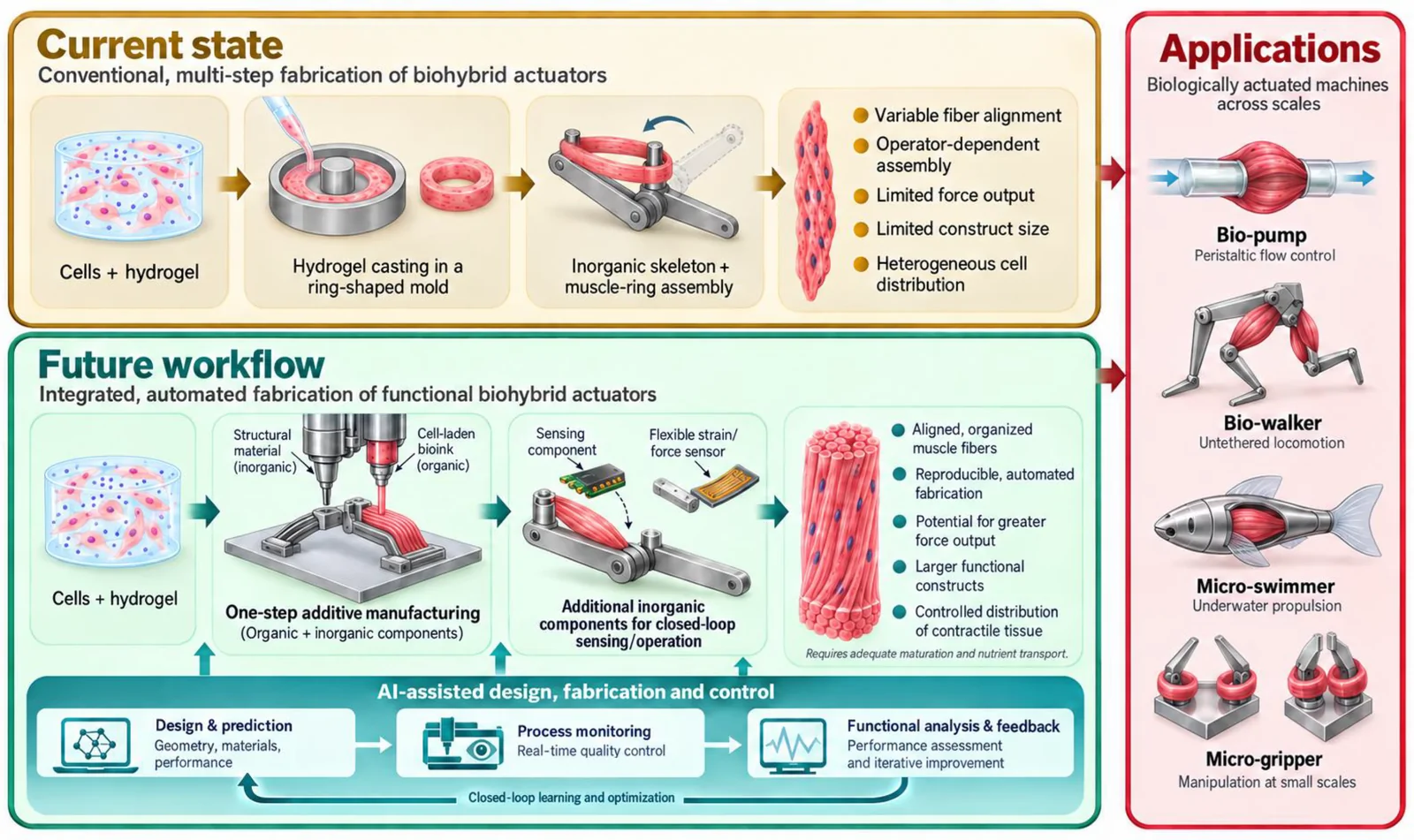
Bioprinting-enabled scaling of biohybrid actuators. Two parallel workflows convert cell-laden hydrogel into a muscle actuator mounted on a synthetic support. The upper workflow illustrates constraints that can arise during fabrication and assembly, including variable alignment, operator dependence, and limited construct size; these are not universal features of casting or electrospinning, both of which can produce aligned tissue under appropriate conditions. Viability shading in both workflows follows the transport model of Section 1: with a representative diffusion distance of 150 µm, a construct of ≈0.3 mm diameter is supplied throughout its cross-section without engineered transport, whereas an unperfused construct of ≈3 mm diameter would retain only about one fifth of its cross-section within diffusion range. The lower workflow therefore shows viable contractile tissue distributed throughout a larger (≈3 mm) anisotropically organised construct only because perfusable channels are printed into it; size and transport are scaled together rather than independently. Artificial intelligence enters at three points: design and prediction, smart fabrication, and post-fabrication analysis. Right: representative application classes. Dimensions are illustrative design targets rather than measured performance limits. Schematic; not to scale.

Bioprinting is a promising approach for engineering of next-generation bioactuators by spatially organizing cells and supporting materials. Under systematic, automated control, bioprinting can reduce operator dependency, enhance functionality, improve structural fidelity, and facilitate the reliable scale-up of biohybrid actuators. These advances may increase force output and contractile performance, thereby advancing biohybrid actuators toward practical robotic deployment (Figure 1) [12].

Although multiple bioactuation strategies exist, skeletal muscle-based actuators are particularly attractive due to their high force generation, adaptability, externally programmable activation, and compatibility with soft robotic architectures. However, scalable fabrication of mechanically integrated skeletal muscle tissues remains an unresolved challenge.

Prior reviews have addressed devices across all cell types [4], system-level advances and biological constraints [6], fabrication techniques for soft and biohybrid actuators [12], and recent trends in muscle integration [13]. The present review focuses on how fabrication choices connect tissue maturation to actuator-level performance. Rather than treating fabrication, stimulation, force transmission, and scalability as separate topics, this review traces how these domains are interdependent, and how advances in bioprinting specifically alter the constraints and opportunities across all of them. In doing so, it aims to provide a coherent engineering perspective on the current state and forward path of muscle-based biohybrid robotics.

## 2. Biological Components for Actuation

Biological components influence the operating range of biohybrid actuators by setting constraints on controllability, force generation, fatigue resistance, and maturation. Two complementary choices must be distinguished. The first is muscle phenotype, skeletal or cardiac, which determines the activation requirements: skeletal muscle requires an external trigger and offers direct control over contraction timing and magnitude, whereas cardiac tissue contracts spontaneously and rhythmically [4,13]. The second is cell provenance, that is, whether the cells are primary isolates, an established immortalized line, or derived from induced pluripotent stem cells. These axes are independent: iPSC derivation is a sourcing strategy applicable to either phenotype [14,15], and it is therefore not a third muscle type to be ranked alongside the first two. Of the thirteen representative systems compiled in Table 1, twelve use skeletal and one cardiac tissue; by provenance, eight use the immortalized C2C12 line and five use primary cells, and none uses iPSC-derived cells. The scalability advantages attributed to iPSC sourcing therefore remain prospective at the actuator level, whatever their standing in tissue engineering more broadly. Together, phenotype and provenance influence achievable force output, temporal dynamics and reproducibility. Table 1 summarizes reported performance benchmarks, fabrication approaches and limitations across representative platforms.

This section compares skeletal and cardiac muscle as contractile components, with particular emphasis on skeletal muscle for externally controlled actuation. It then considers iPSC-derived cells as a complementary sourcing strategy. The comparison addresses force output, controllability, fatigue resistance, maturation, and integration requirements, while recognizing differences in construct geometry and measurement protocols.

### 2.1. Cardiomyocyte-Based Actuation: Autonomy and Endurance

**Figure 2:**
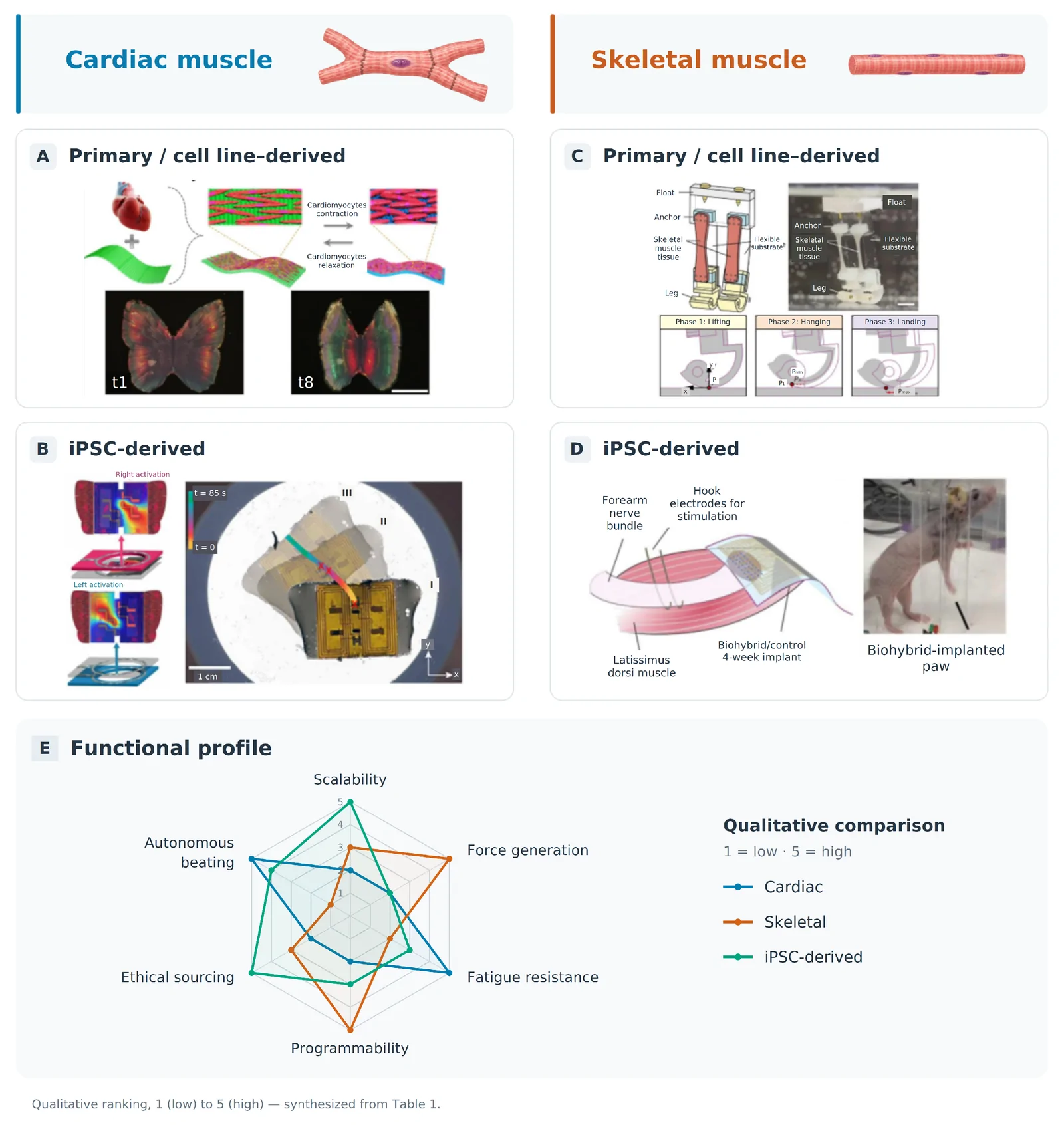
Muscle-powered biohybrid devices from primary and iPSC-derived sources. (**A**) Primary cardiomyocytes cultured on an anisotropic inverse-opal scaffold, producing cyclic structural-colour actuation and butterfly-like flapping (t1, t8) [34]. (**B**) Cardiomyocytes integrated with a wireless bioelectronic interface through electrical synapses with motor neurons, enabling lateralised activation and steerable locomotion at ≈0.52 mm s^−1^ [35]. (**C**) Skeletal muscle tissues anchored to a flexible substrate in a bipedal walker, showing the lifting, hanging and landing phases of the gait cycle [17]. (**D**) iPSC-derived myocytes on a regenerative bioelectronic implant interfaced with host peripheral nerve and recorded over four weeks in vivo [36]. This panel is a neural-interface demonstration rather than an actuator: no contractile output drives a mechanical load. It is included because it illustrates the state of iPSC-derived muscle in this literature, where the reported systems are interfaces and tissue models rather than devices producing measured mechanical work (Section 2). (**E**) Relative comparison of the three cell sources across six performance dimensions; scores are ordinal rankings (1–5) synthesized from Table 1, not measured values. Panel (**A**) reproduced with permission from Fu et al. [34]; copyright 2018, AAAS. Panel (**B**) reproduced with permission from Tetsuka et al. [35]; copyright 2024, AAAS. Panel (**C**) reproduced with permission from Kinjo et al. [17]; copyright 2024, Elsevier. Panel (**D**) reproduced from Rochford et al. [36], licensed under CC BY 4.0. Panel (**E**) is original to this work.

Cardiomyocyte are often selected for biohybrid actuators that require continuous, autonomous motion. Engineered cardiomyocyte cultures can exhibit spontaneous contractions, and when cultured into confluent cardiomyocyte monolayers (CCMs), they tend to form electrically coupled syncytia with high fatigue resistance [8]. The CCMs can maintain contractions for extended periods, with reported peak twitch forces of 10–100 µN in the cited configurations, making them especially suitable for actuators designed for long-term, endurance-based operation. Although absolute force depends on construct geometry and measurement conditions, cardiomyocyte-based bioactuators generate adequate forces for applications that prioritize endurance and autonomous beating over peak contractile strength (see Figure 2A). Moreover, stacking or integrating multiple CCM sheets has been reported to amplify cumulative force output, extending their potential utility [8].

One limitation of cardiomyocytes, however, is that their inherent spontaneous activity can complicate precise control. Continuous beating, while advantageous for endurance, can be a drawback in systems requiring on-demand switching or finely tuned actuation. In such cases, external synchronization methods, such as electrical pacing or optogenetic stimulation, can partially mitigate this limitation by imposing temporal control over contraction. Electrical coupling can synchronize cardiac tissue, but it also complicates independently addressing neighboring regions, making spatial control an important design consideration.

### 2.2. Skeletal Muscle-Based Actuation: Force and Programmability

Skeletal myoblasts used in biohybrid actuators are commonly derived from established cell sources, such as the C2C12 murine myoblast line, which can undergo differentiation and fusion to form contractile multinucleated myotubes. Compared with cardiomyocytes, skeletal muscle cells provide a distinct advantage for robotic applications due to their ability to generate high active forces while remaining externally controllable. Their contractile activity can be precisely regulated through engineered activation strategies, making skeletal muscle particularly attractive for applications requiring programmable and directional mechanical output [8,16]. The mechanisms enabling such controlled activation and their associated engineering challenges are discussed in detail in Section 4 and Section 5.

Engineered skeletal muscle constructs generate measurable contractile forces after differentiation, although reported values vary with construct size, maturity and stimulation regime, and they are best read as individual examples rather than as limits of what the tissue can achieve. All figures below are whole-construct measurements; single-myotube forces are not reported in the systems reviewed here. Under twitch stimulation at 1 Hz, a bipedal walker construct produced 0.61 mN [17] and a bioprinted muscle–tendon unit 300–350 µN at 1 V mm^−1^ [18]. Under tetanic stimulation, the same walker reached 1.9 mN at 50 Hz [17], and individual muscle rings 1.7 mN, rising to 5.9 mN when four were stacked in series [19]. Intermediate values of 400 ± 200 µN [20] and 737–1403 µN [21] have been reported for tethered and walking devices respectively (Figure 2C). Normalized to cross-sectional area, the two studies that report active stress give 0.72 kPa, calculated from a cross-section idealized as a 1.2 × 2.2 mm rectangle [17], and 4.3–4.6 kPa [19]. Neither states the loading mode or the point in culture at which the cross-section was measured, and most studies report no stress at all, so these values support comparison only in the loosest sense; the reporting conventions this would require are set out in Section 4.5. These characteristics distinguish skeletal muscle from spontaneously contracting cardiomyocyte-based systems, where autonomous rhythmic activity is advantageous for some applications but provides less direct control over contraction timing and magnitude. The ability of skeletal muscle tissues to combine high force generation with programmable activation has enabled their integration into biohybrid systems designed for controlled mechanical tasks, including locomotion and manipulation [8,16].

However, skeletal muscle performance is strongly dependent on the engineered cellular microenvironment [8,16]. Scaffold composition, substrate properties, biochemical cues, and extracellular matrix interactions influence myoblast alignment, fusion, sarcomere organization, and the eventual contractile capacity of the tissue [8,16]. Accordingly, researchers have employed natural extracellular matrix components, including collagen, fibrin, and Matrigel, as well as engineered materials with anisotropic architectures, to promote muscle organization and functional maturation [8,16]. These requirements make skeletal muscle-based actuators highly dependent on careful control of their surrounding biomaterial environment and culture conditions, creating both opportunities for engineering optimization and challenges for reproducible fabrication [8,16].

Overall, skeletal muscle should be considered a highly tunable but biologically complex actuator, in which mechanical performance emerges from the interaction between engineered tissue organization and its surrounding environment. This combination of high force output and controllable activation makes skeletal muscle one of the most promising biological engines for biohybrid robotics. However, transforming cellular contraction into functional robotic motion requires additional engineering strategies to efficiently couple biological output with device-level performance, as discussed in Section 4 [8,16].

**Table 1:** Representative muscle-based biohybrid actuators: cell source, matrix, fabrication route, construct scale, reported performance, reported lifetime and principal limitation. Values are as given in the original study; “not reported” indicates that no quantitative value was published, illustrating the inconsistent reporting discussed in Section 4.5. Lifetimes refer to constructs held in culture and actuated intermittently during testing, not to continuous operation. The Cell Type column encodes two independent attributes: muscle phenotype (skeletal or cardiac) and cell provenance (primary isolate, immortalized line, or iPSC-derived).

Cell Type	Materials & Scaffolding	Fabrication Method	Scale	Reported Performance	Reported Lifetime	Actuator Outcomes	Limitations	Ref.
Primary myoblasts (mouse Pax7-nGFP)	Collagen I (3 mg mL^−1^)/fibrinogen (25 mg mL^−1^) photoresin	Filamented light (FLight) bioprinting	Millimeter	Displacement 3% spontaneous, 7% with acetylcholine; force not reported	Not reported	Aligned fibers; Pax7^+^ maintained; self-repair after cardiotoxin injury	Short-term validation; requires optical modulation setup	[22]
Murine myoblast (C2C12)	GelMA/fibrinogen IPN, tunable stress relaxation	Embedded extrusion into Carbopol support bath	Millimeter	Aligned myotube contraction shown; force not reported	Not reported	Faster stress relaxation improved differentiation; cell-free scaffold restored function in rat VML model	Regeneration study, not an actuator; no force reported	[23]
Murine myoblast (C2C12)	GelMA/fibrin/Matrigel, separate muscle and tendon zones	Extrusion-based multicellular bioprinting	Millimeter	Peak force 300–350 µN (1 Hz, 1 V mm^−1^); displacement 100–200 µm	Function retained 3 months in culture	Muscle–tendon unit with improved force transmission	Force below the mN range needed for practical tasks	[18]
Murine myoblast (C2C12)	Fibrin/collagen I/Matrigel strip; PEGDA ($M_{r}$ 700) skeleton	SLA-printed skeleton; cast muscle strip	Millimeter	Locomotion 156 µm s^−1^ (4 Hz); active tension 394.7 ± 56.6 µN	Not reported	Asymmetric two-pillar bio-bot walks under electrical stimulation	Low passive stress (0.84 kPa); direction set by pillar asymmetry	[24]
Human skeletal myoblasts	Fibrinogen/Matrigel; PDMS anchors	Custom PDMS ring molding	Millimeter ring, Centimeter robot	Tetanic 1.7 mN per ring (5.9 mN for four stacked); stress 4.3–4.6 kPa	Not reported	PDMS ring resists tearing during compaction; multijoint robot driven by tetanus	Manual casting; low throughput	[19]
Primary neonatal rat cardiomyocytes	CNT–GelMA (5 wt%, 1 mg mL^−1^ CNT) on patterned PEG	Layered casting; embedded Au microelectrodes	Millimeter	Force not reported; beating rate controlled to 1.0 Hz below 3 V	Not reported	Stingray robot; localized control via integrated microelectrodes	No propulsion shown; wireless operation proposed, not implemented	[25]
Optogenetic murine myoblast (C2C12)	Fibrin/Matrigel tissue on linear-elastic flexures	Cast onto rigid–flexible flexures	Millimeter	Force not reported; stroke ≈5× prior values; tetanus at 4 Hz	Not reported	Flexures amplify and decode contraction without sensors	Strain ≈1% of length vs. ≈20% in native muscle	[26]
Murine myoblast (C2C12)	Parylene-C-coated printed resin struts and thread; fibrinogen/Cultrex BME tissue	Cast tissue snap-fitted to printed tensegrity skeleton	Centimeter	Contraction ≈ 0.5 mm (>3% of 13 mm length) at ≥4 V mm^−1^; force not stated	Force peaks day 10, declines by day 12	Selective contraction of three tissues gives 3D multi-DOF tilting	Manual assembly; no locomotion shown; C2C12 contractility limits output	[27]
Murine myoblast (C2C12)	PDMS serpentine spring skeleton; fibrinogen/thrombin/Matrigel	Extrusion-printed PDMS skeleton; cast hydrogel	Millimeter	Swimming 800 µm s^−1^ (3 body lengths s^−1^) at 5 Hz; force not measured	Not reported	Spring restoring force self-trains the tissue without external stimulation	Force only simulated; asymmetric stiffness needed for direction	[28]
Optogenetic murine myoblast (C2C12)	PEGDA skeleton (20%, E≈ 270 kPa); fibrinogen/thrombin/Matrigel	DLP-printed skeleton; cast muscle ring	Centimeter	Walking 0.83 mm s^−1^ (4 Hz, 50 ms pulse); muscle force 737–1403 µN	2–3 weeks with ACA; tested days 15–21	Battery-free wireless optogenetic control; 10× speed gain over three iterations	Speed limited by muscle active tension and scaffold compliance	[21]
Primary human myoblast (HSkMC)	Fibrinogen/Geltrex tissue; parylene-C pillar anchor filled with low-melting-point alloy	SLA anchor, vacuum-filled alloy pillars; cast tissue	Millimeter	Contractile distance 108–165 µm (15–45 Vpp); 178 vs. 8 µm target vs. neighbour at 4 mm	20-day culture; peaks day 12	Embedded pillars: selective index 0.94 vs. 0.33 for external Au rods	Complex alloy-pillar fabrication (75% success); force not quantified	[29]
Primary neonatal rat myoblasts	PDMS substrate with anchors; fibronectin/Matrigel	Cast on PDMS substrate; inkjet-printed legs	Millimeter tissue, Centimeter robot	Twitch 0.61 mN (1 Hz); tetanic 1.9 mN (50 Hz); stress 0.72 kPa	Not reported	Bipedal robot: 5.4 mm min^−1^; 90° turn in 62 s	Electrodes positioned manually; operates only when submerged	[17]
Optogenetic murine myoblast (C2C12)	PVA:PAA IPN tendon on polyurethane backing; micro-milled LDPE flexures	Cast muscle in PDMS molds; molded, laser-cut tendons	Millimeter	≈11× power-to-weight vs. earlier designs, same muscle; force 400 ± 200 µN; interface >5 mN	Not reported	Tendon transmits force without delamination; modular actuators	No stiffness gradient at the interface; immortalized line, not primary human cells	[20]

### 2.3. iPSC-Derived Muscle Cells: Cell Sourcing and Standardization

The growing use of induced pluripotent stem cells (iPSCs) for cardiomyocyte generation in biohybrid actuator research is driven by several complementary advantages. One of the most important advantages is the ability to obtain large numbers of contractile cells from readily accessible somatic sources, which removes the dependence on freshly harvested primary tissue that constrains how many constructs can be produced from a single isolation [8,15]. Because iPSCs can be expanded extensively before differentiation, they can supply far more cells per derivation than primary isolation allows. This scalability should not be read as standardization or as an unambiguous ethical simplification. Donor consent and the terms under which somatic cells were obtained remain live questions for any cell-derived engineered construct [30], and reproducibility is limited in practice by line-to-line variation between iPSC clones; by the composition of the differentiation media and supplements used, several of which remain animal-derived; and by batch-to-batch variability in differentiation efficiency. A scalable source is therefore not automatically a defined one. Although iPSC-derived cardiomyocytes generally produce lower contractile forces than primary cardiomyocytes, advances in biochemical conditioning, electrical and mechanical stimulation, and scaffold design have substantially improved their maturation and long-term functional stability. These developments have made them increasingly suitable for incorporation into biohybrid devices [15].

By contrast, differentiating iPSCs into mature skeletal myotubes remains considerably more demanding. Skeletal myogenesis requires the coordinated activation of multiple signaling pathways, including Wnt, Notch, and IGF-1/mTOR, together with precise temporal control and appropriate spatial organization. These conditions are necessary to reproduce the formation of aligned, multinucleated myotubes with well-developed sarcomeric structures [14]. As a result, iPSC-derived skeletal muscle constructs often remain relatively immature and still show limited force generation and tissue integrity compared with native skeletal muscle [8,14,31].

This difference in differentiation complexity and maturation efficiency has encouraged the broader adoption of iPSC-derived cardiomyocytes, for which protocols are currently more standardized, reproducible, and scalable in biohybrid actuator applications (Figure 2B). iPSC-derived systems therefore introduce a sourcing–maturation trade-off: they offer strong advantages in scalability and sourcing, but their functional performance remains constrained by incomplete maturation and variable force output. Taken together, these cell sources define a multidimensional trade-off landscape for biohybrid actuation. Cardiomyocytes are well-suited to applications requiring autonomous rhythmic activity and endurance, iPSC-derived systems offer expandable cell sourcing, and skeletal myotubes support externally controlled force generation. Within this framework, skeletal muscle remains a particularly promising substrate for bioprinted biohybrid robotics, provided that scaffold design can adequately control the microenvironmental conditions required for stable maturation and sustained mechanical performance.

### 2.4. Selecting the Cell Source for the Intended Robotic Function

The comparison of muscle phenotypes and cell-sourcing strategies demonstrates that no single biological actuator is universally optimal for all biohybrid robotic applications. Instead, the choice of cell source should be guided by the functional requirements of the intended robotic system, as each muscle type occupies a distinct region of the actuator design space defined by force generation, controllability, endurance, and manufacturability. Consequently, selecting a biological actuator is an engineering design decision in which biological characteristics are matched to the robot’s desired mechanical and functional performance rather than maximizing a single performance metric [6,32].

Cardiomyocytes are particularly well suited to applications requiring continuous rhythmic motion without externally timed stimulation. Spontaneous activity within coupled cultures supports sustained rhythmic actuation, making them attractive for swimming robots, micropumps, and other systems that benefit from sustained cyclic contractions. However, because contraction originates from spontaneous electrical synchronization throughout the tissue, independent temporal or spatial control of actuation remains limited. As a result, cardiomyocyte-based actuators are generally preferred when continuous operation is more important than precise motion control [32,33].

In contrast, skeletal muscle supports programmable actuation. Because contractions can be triggered by external stimulation, both the timing and magnitude of force generation can be regulated with high precision. When combined with sufficient contractile output, this supports directional locomotion, grasping, manipulation, and other robotic behaviors that require controlled mechanical output. These characteristics have consequently established skeletal muscle as the predominant biological actuator for biohybrid robots designed to perform complex, task-specific movements under external control [6].

iPSC technology provides an expandable source of cells that can be differentiated toward either cardiac or skeletal muscle. Its principal advantage lies in cell sourcing and the potential for standardized production, rather than in a distinct mode of actuation. Achieving reproducible performance nevertheless requires control of differentiation efficiency, maturation, and variation between cell lines and production batches [14,15].

Taken together, these considerations indicate that the biological actuator should be selected according to the primary objective of the robotic platform. Cardiomyocytes are advantageous for autonomous, long-duration rhythmic actuation, whereas skeletal muscle is preferable for high force generation and precise on-demand control. iPSC-derived muscle cells provide a complementary strategy when reproducibility, scalability, and standardized cell sourcing become primary design considerations (Figure 2B,D). Since most biohybrid robotic systems developed for locomotion, object manipulation, and programmable actuation require accurate temporal control together with relatively high mechanical output, skeletal muscle currently represents a versatile biological actuator for engineered biohybrid robots and therefore serves as the principal focus of this review [8,32].

## 3. Engineering the Muscle Actuators

Scaling up the design of biohybrid muscle actuators relies on the interplay of multi-scale architectural strategies, where soft, biocompatible, extracellular matrix (ECM)-like biomaterials provide the microenvironment required for muscle development and function, while integrated structural materials ensure mechanical robustness and preserve coordinated contractile activity across the construct [4,37]. Achieving this level of integration remains highly challenging but is increasingly enabled by advances in additive manufacturing, including high-resolution biofabrication with feature sizes approaching tens of micrometers, on-demand multimaterial deposition, and the ability to spatially organize materials with distinct biological and mechanical properties within a single construct [24,32,38]. Nevertheless, fabrication technologies alone do not ensure functional scalability. The performance of muscle-based bioactuators ultimately depends on the coordinated engineering of tissue architecture, mass transport, mechanical loading, electrical accessibility, and force-transfer interfaces, all of which remain tightly coupled across multiple length scales. Accordingly, this section discusses the engineering of skeletal muscle actuators from their fundamental architectural requirements through emerging biofabrication strategies, biomaterial selection, structural reinforcement, and conductive functional materials that collectively determine actuator-level performance.

### 3.1. Architectural Requirements of Contractile Tissues

Skeletal muscle is a highly organized tissue whose contractile performance depends on the coordinated interplay between cellular organization, biochemical microenvironment, and mechanical cues [37,39]. Consequently, engineering muscle-based bioactuators extends beyond maintaining cell viability and requires the recreation of structural features that support efficient tissue organization, maturation, and force generation [4,37]. From an engineering perspective, skeletal muscle actuators must therefore satisfy a set of interdependent architectural requirements that collectively influence tissue development, contractile performance, and scalability [4,40].

A primary requirement of engineered skeletal muscle is the reproduction of the anisotropic organization characteristic of native muscle tissue. The contractile apparatus relies on highly aligned multinucleated myofibers, in which cellular orientation enables directional forces and coordinated contraction [37,41]. The parallel alignment of muscle cells promotes sarcomere organization and improves the efficiency of force generation, making anisotropic tissue architecture a fundamental design objective for functional muscle constructs [41,42]. Accordingly, biofabrication approaches increasingly aim to control cellular alignment through structural guidance cues, patterned substrates, and engineered microarchitectures that promote myogenic maturation and organization [41,42].

In addition to cellular organization, engineered muscle tissues must maintain sufficient transport of oxygen, nutrients, and metabolic waste throughout the construct, making mass transport an essential consideration in three-dimensional tissue design [10,40]. Diffusion alone limits a cast construct to roughly the scale at which oxygen can reach its centre, so thickness beyond that point requires transport structures built into the tissue rather than simply more material. Printed constructs containing embedded perfusable channels have been shown to sustain cell viability at thicknesses where unperfused equivalents fail [11]. The coupling between mass transport, fiber-scale anisotropy, and local mechanical properties collectively regulates myocyte viability, differentiation, and the formation of organized contractile units [11].

Beyond cellular and structural organization, reproducible architectural control is essential for the reliable fabrication and evaluation of engineered muscle tissues. Parameters such as tissue geometry, alignment, and material distribution influence differentiation outcomes and contribute to variability among constructs, highlighting the importance of precise control over fabrication parameters [40,43]. Such architectural consistency is particularly important for translating engineered muscle tissues from biological models into functional biohybrid actuators, where variations in construct design can directly influence measured performance.

Collectively, these architectural requirements define the design principles necessary for constructing functional skeletal muscle tissues. However, achieving robotic functionality requires additional engineering strategies that connect tissue-level contractility with actuator-level performance, including stimulation, mechanical integration, and force transmission, as discussed in Section 4. The following section describes how emerging additive manufacturing and biofabrication approaches provide control over tissue organization, geometry, material distribution, and structural complexity to address these architectural challenges.

### 3.2. Additive and Biofabrication Strategies

Additive manufacturing provides a powerful platform for engineering skeletal muscle bioactuators by enabling precise control over tissue geometry, cellular organization, and spatial distribution of biological and structural components. While the direct application of 3D bioprinting to bioactuator fabrication remains in its infancy, additive manufacturing has already demonstrated substantial potential to replicate the architecture and functionality of skeletal muscle and cardiac tissues in tissue engineering applications [40]. These approaches provide a transferable framework for biohybrid actuators, where contractile muscle constructs must be integrated with supporting architectures to generate controlled mechanical output.

Additive manufacturing enters biohybrid actuator fabrication at two distinct points, and the distinction is often blurred. In the more common arrangement, printing produces the structural element—a skeleton, anchor, joint or limb—onto or into which separately prepared muscle tissue is then cast. Stereolithographic and digital-light-processing PEGDA skeletons [21,24], extrusion-printed elastomeric springs [28], printed tensegrity frames [27], inkjet-printed limbs [17] and printed anchor structures [29] all follow this route: the printer never touches a cell. In the second arrangement, the contractile tissue itself is the printed object, with cells suspended in the ink and deposited in a defined architecture. Filamented-light bioprinting of aligned myofibres [22], embedded extrusion of myoblast-laden interpenetrating networks [23] and multicellular printing of spatially separated muscle and tendon zones within a single construct [18] belong to this second category. Of the thirteen representative systems compiled in Table 1, six are printed structures with cast tissue; three are directly bioprinted tissues; and in the remaining four, the load-bearing elements are moulded or machined rather than printed. This distribution matters for how performance should be read: printing the skeleton improves geometric control over the actuator, whereas printing the tissue is what offers control over the internal architecture on which force generation depends, and only the second addresses the maturation-limited specific forces discussed in Section 4.5. Table 1 accordingly separates the tissue-level force a construct generates from the device-level motion it produces, since a printed skeleton can improve the latter without changing the former. Bioprinted constructs developed for volumetric muscle loss repair illustrate the second route in a regenerative rather than robotic setting [23].

Among available approaches, extrusion-based bioprinting is a widely used strategy for fabricating engineered skeletal muscle constructs due to its ability to process large volumes of cell-laden biomaterials with cell viability dependent on processing conditions [41]. Furthermore, the shear forces generated during extrusion can induce anisotropy within bioink components, promoting preferential alignment of polymer chains, incorporated fibers, and suspended muscle progenitors [42]. By controlling parameters such as nozzle diameter, extrusion pressure, flow rate, the fiber aspect ratio, and ink viscosity, the degree of alignment and structural organization can be tuned to influence the final architecture of engineered muscle tissues [44].

The ability of extrusion-based approaches to generate aligned architectures is particularly relevant for muscle bioactuators, where directional organization is essential for efficient contraction and force generation [41,42]. However, the shear conditions required to achieve alignment must be carefully balanced, as excessive hydrodynamic stresses can compromise cell viability and negatively affect tissue maturation. Therefore, extrusion-based fabrication requires optimization of both material properties and printing parameters to achieve reproducible anisotropic constructs while preserving biological function [42].

Compared with bulk hydrogels produced by casting, three-dimensional bioprinted constructs provide improved control over tissue architecture by enabling defined filament spacing, alignment, built-in anchors, electrodes, and perfusable channels [29,40]. These capabilities allow the generation of reproducible geometries and spatially resolved stimulation and readout strategies, improving comparability between engineered tissues and facilitating functional characterization [43].

**Figure 3:**
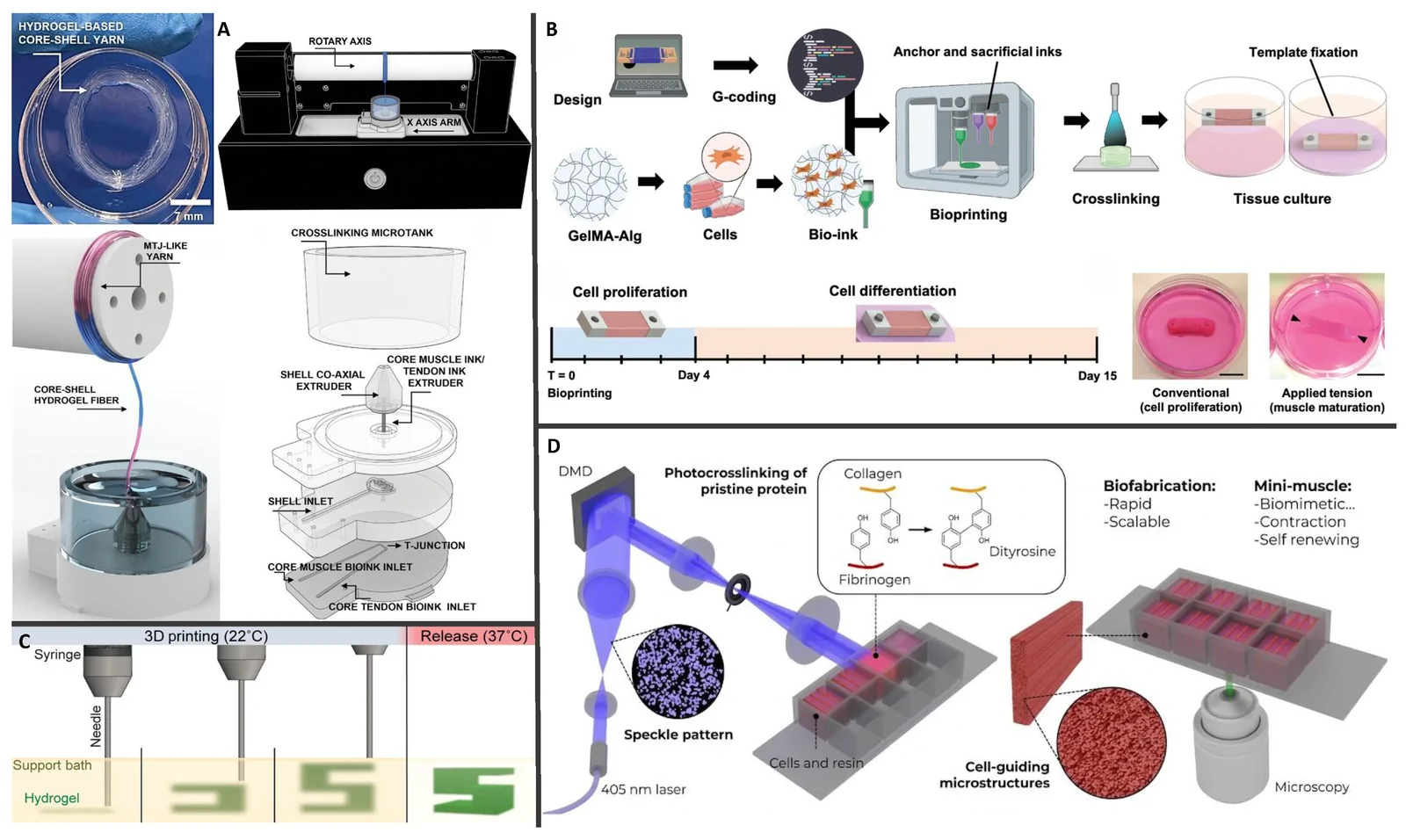
Emerging fabrication approaches for biomimetic muscle tissues. (**A**) Rotary wet-spinning platform producing core–shell hydrogel fibres with distinct muscle and tendon compartments [45]. (**B**) Extrusion pipeline from design and G-coding through GelMA–alginate bioink preparation, printing and crosslinking to tissue culture [47]. (**C**) Embedded printing within a support bath at 22 °C, with thermal release of the construct at 37 °C [49]. (**D**) Filamented light bioprinting using dityrosine crosslinking of collagen and fibrinogen to produce cell-guiding microstructures and self-renewing mini-muscles [22]. Panels (**A**–**D**) are reproduced from Volpi et al. [45], Filippi et al. [47], Hinton et al. [49] and Liu et al. [22], respectively, and are licensed under CC BY 4.0.

Beyond conventional extrusion systems, microfluidics-assisted biofabrication couples the ink streams upstream of the nozzle, so that composition can be varied along a single filament and heterogeneous architectures assembled from more than one bioink [41]. Microfluidics-assisted three-dimensional rotary wet spinning (RoWS) applies this principle to fibrous tissues: a microfluidic head extrudes a core–shell filament, which a rotating drum collects into densely packed, aligned bundles [45]. The rapidly crosslinked shell allows low-viscosity, cell-permissive inks to be used in the core, which would not otherwise hold a filament, and the resulting fiber alignment supports myogenic differentiation. Fabrication is rapid rather than high-throughput in any quantified sense: the authors report meter-long fibers produced in a short time, with a bundle of twenty core–shell fibers corresponding to approximately thirty seconds of extrusion. The approach has been used to build myotendinous-junction-like yarns in which tendon-like and muscle-like regions meet within a single construct, and these yarns were shown to possess a stiffness intermediate between the two constituent tissue types (Figure 3A) [45]. Improved mechanical coupling across the interface is the intended benefit, but it remains an intended rather than a demonstrated outcome: the study reports no active force measurement and no comparison of transmitted force against a non-graded control, so the mechanical grading is established while its functional consequence is not.

Beyond these approaches, multimaterial fabrication represents a key strategy for integrating the diverse biological and mechanical requirements of muscle bioactuators within a single construct. The spatial deposition of ECM-like biomaterials, structural components, and functional materials enables the creation of complex architectures that better recapitulate the hierarchical organization of native muscle tissues [46]. For example, centimeter-scale biohybrid skeletal muscle constructs have been fabricated through extrusion-based multimaterial bioprinting of GelMA–alginate blends combined with sacrificial Pluronic F127 inks to generate perfusable microchannel networks. These integrated architectures enhance cell viability, promote structural organization, and enable functional applications including mechanical stimulation studies and drug distribution assays (Figure 3B) [47]. Multimaterial printing also enables spatial control over tissue composition at the muscle–tendon interface: co-printing myogenic and fibroblast-laden bioinks into defined zones, joined by an interdigitated interface layer, produces bioactuators with greater contractile displacement than single-material full-muscle controls, while the stiffness gradient between muscle and tendon regions disperses contraction-induced focal stresses across the junction. Actuators fabricated this way retained contractile function over a three-month culture period [18].

While extrusion and microfluidic approaches provide control over aligned architectures, embedded bioprinting addresses a different constraint: the inability of low-viscosity inks to hold a shape while they are being deposited. The formulations that best support myogenesis are precisely the problematic ones, since fibrinogen, Matrigel and decellularized-matrix inks lack the yield stress needed to sustain a complex geometry unsupported [48]. Embedding the nozzle in a surrounding medium removes that requirement, and two strategies do so in opposite ways. In freeform reversible embedding of suspended hydrogels (FRESH), the construct is written into a sacrificial gelatin-slurry bath that mechanically supports each deposited filament and is then melted away, leaving the printed object [49]; the approach has since been applied to cardiac tissue for in vitro pharmacology [43]. In sacrificial writing into functional tissue (SWIFT), the relationship is inverted: the printed ink is itself sacrificial and is written into a dense living matrix of cellular building blocks, which is the product, and its removal leaves a perfusable channel network behind [50]. The two are therefore not interchangeable, since FRESH extends the range of printable inks, whereas SWIFT solves a transport problem in tissue that is already dense (Figure 3C). Both remain generic platform capabilities in the present context: FRESH and SWIFT have been demonstrated on collagen and cardiac tissue rather than on muscle-powered actuators, and the only embedded-printing study of skeletal muscle among those compiled here used extrusion into a Carbopol support bath for volumetric muscle loss repair, without reporting contractile force [23]. The translation of embedded printing into a working biohybrid actuator has not yet been reported.

In parallel, light-based fabrication approaches have expanded the ability to generate microscale architectures with high geometric precision. Advanced photopolymerization techniques, including Filamented Light (FLight) printing, enable the organization of low-viscosity bioinks into highly defined anisotropic structures suitable for myogenic differentiation (Figure 3D) [22,51]. By exploiting optical modulation instability to generate multiple self-focused micro-beams, FLight enables the photopolymerization of cell-compatible hydrogel architectures with microscale resolution and controlled anisotropy. FLight addresses the same low-viscosity processing problem as RoWS, FRESH, and SWIFT, but by a fundamentally different route: whereas RoWS and FRESH rely on physical confinement, either a coaxial shell or a support bath, to hold the material in place, FLight organizes the bioink optically during polymerization. This produces microfilament arrays at a finer scale than confinement-based methods achieve, yielding fascicle-like organization directly rather than through subsequent maturation, at the cost of a construct thickness limit imposed by light penetration [22,51].

These strategies are not interchangeable, and Table 2 shows that they occupy different positions on three separable axes: whether tissue contraction has been measured, whether that contraction has been transmitted to a load-bearing structure, and whether an assembled device has locomoted. The distinction matters because the three do not move together. Casting onto synthetic skeletons and micropatterned substrates satisfy all three, but both depend on operator-dependent tissue handling. Extrusion bioprinting has produced a bioprinted muscle–tendon unit generating 300–350 µN and transmitting it through an engineered tendon to anchors [18], satisfying the first two without yet driving a locomoting device. Filamented light bioprinting has produced contractile mini-muscles with measured displacement but no reported force and no structural integration [22]. In two further cases, the locomotion belongs to the cast tissue rather than to the printed component: vat photopolymerization supplies the skeleton, and drop-on-demand printing supplied the synthetic legs, in devices whose muscle was cast [17,21,24]. Attributing device-level performance to the fabrication method that made only the structural part overstates what that method has shown. The pattern across Table 2 is therefore not a fixed trade-off between automation and performance but an incomplete overlap: the automated routes have begun to produce measurable contraction and transmitted force, while integration, assembly and quality control remain manual. Whether this is a persistent constraint cannot be determined from the present evidence, since no study compiled here reports a fabrication throughput figure against which automation could be assessed. The unresolved issue is how to combine automated deposition with validated force transmission, assembly and quality control in a single production workflow.

**Table 2:** Comparison of major biofabrication strategies used for engineering muscle-based biohybrid actuators. Demonstrated outcomes are reported at three separable levels: measured tissue contraction, load-bearing actuation against a structure, and locomotion of an assembled device. A strategy may achieve one without the others, and in some entries, the locomotion is produced by cast tissue carried on a printed structure rather than by printed tissue; the level reported is what the strategy has itself produced, not what it is in principle capable of. Scalability is an assessment rather than a measurement, since no study compiled here reports a fabrication throughput figure. Contractile values are cross-referenced to Table 1.

Fabrication Strategy	Typical Resolution	Cell Viability	Key Advantages	Typical Applications	Demonstrated Outcomes	Scalability Potential	Ref.
Extrusion bioprinting	∼100–200 µm	High	Compatible with a wide range of bioinks and suitable for large-scale tissue constructs; support-bath variants extend the usable range to low-viscosity inks.	Engineered skeletal muscle tissues and volumetric muscle loss repair.	*Contraction:* 300–350 µN, 100–200 µm displacement. *Load-bearing:* yes, via a printed muscle–tendon unit. *Locomotion:* not demonstrated.	High (automated deposition compatible with large construct volumes and multi-material printing)	[18,23,48,52]
Multi-material conductive extrusion	∼100–200 µm	High (87%)	Co-deposition of cell-laden and piezoresistive inks within a single construct; printed interfaces outperform cast equivalents in viability and sensor–tissue integrity.	Integrated sensing and stimulation architectures.	*Contraction:* not reported; ink formulation and sensing characterized only. *Load-bearing:* no. *Locomotion:* no.	Moderate (multi-ink alignment and rheological matching limit throughput)	[53]
Drop-on-demand bioprinting	∼50–100 µm	High (method); not applied to muscle cells to date	Precise cell deposition and spatial patterning; encompasses inkjet, electrohydrodynamic, acoustic and microvalve modalities.	Patterned tissue constructs; structural components of biohybrid devices.	*Contraction:* not reported; no muscle tissue has been printed by this route. *Load-bearing:* not by this route. *Locomotion:* in the one biohybrid device using it, inkjet printing formed the synthetic legs and the muscle was cast.	Moderate (high precision but low throughput limits large-scale construct fabrication)	[17,44]
Vat photopolymerization (SLA/DLP)	∼25–100 µm	N/A—structural parts only; tissue cast separately	High structural precision and rapid fabrication of complex geometries.	Synthetic skeletons for hybrid robotic structures.	*Contraction:* not applicable; the method produces the skeleton, not the tissue. *Load-bearing:* yes, via cast muscle on a printed skeleton. *Locomotion:* yes—walking and swimming devices [21,24].	Moderate (rapid fabrication, but the tissue must still be cast and assembled manually)	[21,24,27]
Filamented light bioprinting	∼3–6 µm microfilaments	High	Enables the fabrication of aligned fibrous structures that support muscle maturation.	Regenerative muscle constructs.	*Contraction:* 3% spontaneous and 7% acetylcholine-evoked displacement; force not reported. *Load-bearing:* no. *Locomotion:* not demonstrated.	Low-Moderate (early-stage technology; throughput and scalability not yet established)	[22]
Casting with synthetic skeletons	N/A	Very high	Simple fabrication process and robust tissue anchoring; the skeleton may itself be printed, so only the tissue step is manual.	Biohybrid swimmers, walkers, grippers and actuator platforms.	*Contraction:* quantified force output across multiple devices. *Load-bearing:* yes. *Locomotion:* yes—swimmers, walkers and grippers [19,21,28].	Low (operator-dependent tissue assembly; limited reproducibility and automation)	[19,21,25,26,28,29]
Micropatterned substrates (soft lithography)	∼20 µm line width	High	Microcontact-printed ECM protein patterns guide cellular alignment and anisotropic myogenesis without a supporting scaffold.	Thin-film actuators and soft robotic devices.	*Contraction:* specific force up to 4 kPa. *Load-bearing:* yes. *Locomotion:* yes—centimeter-scale muscular thin films gripping, pumping, walking and swimming [54].	Low (manual fabrication process; restricted to quasi-two-dimensional tissue)	[4,54]

This distinction also matters when comparing reported construct sizes, because the largest printed construct and the largest functional actuator are not the same object. Centimetre-scale muscle constructs have been produced by extrusion-based multimaterial printing with sacrificial perfusion channels [23,47], but their dimensions must be interpreted alongside the specific viability, force, and device-level outcomes measured. Several devices with quantified force and locomotion use millimeter-scale muscle tissues fabricated by casting or molding. The bipedal walker of Kinjo et al., for example, generates 1.9 mN of tetanic force at 50 Hz, from which the authors estimate a contractile stress of 0.72 kPa (reported as 0.72 mN mm^−2^) using a cross-section idealized as a 1.2 × 2.2 mm rectangle; the report does not state when these dimensions were measured relative to the force test [17]. The ring-based multijoint robot of Morita et al. produces a comparable 1.7 mN per ring, but at 4.3–4.6 kPa [19]. The two raw forces appear equivalent, while the specific forces differ approximately sixfold, because the cross-sections differ; force values are therefore not comparable across systems unless the normalizing area and the basis on which it was obtained are both stated, as set out in Section 4.5. Pillar-electrode systems have demonstrated increased contractile displacement over the same scale range but report no force, and so cannot be placed on this comparison at all [29]. Demonstrating force and useful motion throughout larger constructs, rather than printing larger volumes alone, is the relevant scaling goal. Among the automated approaches, the appropriate choice depends on which architectural constraint dominates. Extrusion is attractive where construct volume and bioink flexibility matter most, and its shear-induced alignment is a design advantage rather than an incidental effect, provided the shear window that aligns without compromising viability is respected. Embedded approaches provide complementary capabilities: support baths can stabilize printed tissue geometries, while sacrificial writing can introduce transport channels within dense living matrices. Light-based methods offer fine anisotropic control at the fascicle scale, but depend on light penetration through the photopolymer and are therefore constrained in construct thickness. For centimeter-scale actuators intended for eventual manufacture, extrusion-based multimaterial printing combined with sacrificial channel writing offers one promising route, whereas embedded and light-based methods can address specific architectural features such as muscle–tendon interfaces and fascicle-level alignment [45,47,50,51].

### 3.3. Biomaterials for Cell Maturation and Contractile Function

Following the selection of an appropriate fabrication strategy, the choice of biomaterials largely determines muscle maturation, structural organization, and long-term contractile function [37,39]. The extracellular matrix (ECM) not only provides structural support but also regulates myoblast alignment, differentiation, and force generation, making biomaterial selection a central design parameter for engineered skeletal muscle actuators [37,39]. Accordingly, biological matrices should provide an appropriate microenvironment while also meeting the mechanical and processing requirements of biofabrication [40,41].

Among available matrices, basement membrane extracts such as Matrigel^®^, Geltrex^®^, and Cultrex^®^ are widely used because their matrix components can support myoblast fusion, alignment, and sarcomere organization [27,29,55]. However, their undefined composition, batch-to-batch variability, animal-derived origin, and rapid thermal gelation complicate bioprinting and large-scale standardization [48,52]. Alternative biomaterials, including fibrin, collagen, GelMA, alginate, and decellularized ECM, have therefore been explored to address these limitations, each offering distinct trade-offs between biological performance, printability, and reproducibility, as summarized in Table 3.

These biomaterials offer different balances of printability, biological activity, and reproducibility. Basement membrane extracts support myogenic culture but introduce compositional variability and processing constraints. GelMA and GelMA–alginate blends offer tunable crosslinking and mechanical properties, although their support for myogenesis depends on formulation and processing. Fibrin and collagen provide biologically relevant matrices but may require stabilization to maintain long-term structural integrity. Tissue-derived dECM can retain useful biochemical cues, while its properties depend on tissue source and decellularization. The stiffness values in Table 3 should be read with the same caution: they are drawn from studies using different measurement modes, and the shear storage modulus and Young modulus for the same gel differ by roughly a factor of three, which is comparable to some of the differences between materials. Nor can contractile output be attributed to any single component, since these matrices are used in the cited work as multi-component blends that no study has varied one at a time. Material selection should therefore be matched to the fabrication method and validated against cell maturation, contractile output, and batch reproducibility rather than based on a universal ranking.

**Table 3:** Materials used as scaffolding matrices and structural elements in muscle-based biohybrid actuators. Stiffness values are indicative rather than matched: across the cited literature, they combine shear storage with Young’s and compressive moduli, which for an incompressible hydrogel differ by about a factor of three ($E\approx 3G^{\prime }$), and measurement conditions are seldom reported. Contractile output cannot be attributed to any single material, since every construct in Table 1 uses a multi-component blend. type n.s. = modulus type not stated in the cited source.

Material	Origin	Reported Stiffness	Printability	Myogenic Support	Batch Variability	Key Limitations	Refs
Matrigel/Geltrex/Cultrex	Animal-derived basement membrane (laminin, collagen IV, growth factors)	0.1–0.4 kPa (type n.s.; 30–60% *v*/*v*; thermal, 37 °C)	Poor (thermal gelation limits deposition precision)	Excellent (fusion, alignment, sarcomere organization)	High	Animal-derived; not scalable; rapid gelation incompatible with bioprinting; lot-dependent growth-factor content drives between-batch variation	[27,55,56]
Fibrin	Fibrinogen + thrombin (human/animal)	0.1–1 kPa (type n.s.; 4–20 mg mL^−1^; thrombin)	Moderate (fast gelation, low viscosity)	Good (myoblast adhesion and differentiation)	Moderate	Fast degradation; low mechanical stability; requires antifibrinolytic agents (aminocaproic acid in essentially all reported constructs)	[19,21,29]
Collagen type I	Animal-derived ECM protein	0.02–0.5 kPa (type n.s.; 1–3 mg mL^−1^; thermal/pH)	Moderate (slow gelation; low shape fidelity)	Good (native adhesion ligands)	Moderate	Low stiffness; slow crosslinking; batch variability	[22,56]
GelMA	Photocrosslinkable modified gelatin	1–100 kPa (type n.s.; 5–20% *w*/*v*; UV, dose-dependent)	Excellent (extrusion and DLP compatible)	Good (retains gelatin adhesion motifs)	Low	Requires UV exposure; photoinitiator cytotoxicity at high doses	[18,47]
Alginate	Seaweed-derived polysaccharide	1–100 kPa (type n.s.; 1–5% *w*/*v*; ionic, Ca^2+^)	Excellent (fast ionic crosslinking; sacrificial or structural ink)	Poor alone (no adhesion motifs; requires RGD modification)	Low	Poor myogenic support without functionalization; ionic crosslinks are reversible and dissolve in chelating agents	[56,57]
GelMA–alginate blend	Blend of modified gelatin and polysaccharide	Not reported as a single value; spans both ranges, set by blend ratio and the two crosslinking steps	Excellent—ionic pre-gelation holds the filament while GelMA is photocured, decoupling printability from stiffness	Good—GelMA supplies the adhesion motifs alginate lacks	Low	Two chemistries must be balanced; residual alginate restricts cell spreading and matrix remodelling	[56,57]
dECM (decellularized ECM)	Tissue-derived (skeletal or cardiac)	Not reported; source- and digestion-dependent	Moderate (requires formulation; low viscosity)	Excellent (retains native biochemical cues)	High	Expensive; laborious preparation; batch-to-batch variability	[48,56]
PEGDA	Fully synthetic photopolymer	≈200–750 kPa (*E*; 20% *w*/*v*, $M_{r}$ 700; UV)	Excellent (SLA and DLP; highest geometric fidelity listed)	None alone—bioinert; used as the cast-on skeleton	Very low	No adhesion motifs; non-degradable. Skeleton stiffness becomes a design variable the tissue must work against rather than a material property	[21,24,58]

### 3.4. Conductive and Stimuli-Responsive Materials

Beyond conventional hydrogel systems designed primarily for structural support and cellular organization, functional materials with conductive or stimuli-responsive properties offer additional opportunities to engineer advanced biohybrid actuator platforms. These materials can provide enhanced compatibility with integrated electronic components and enable the development of multifunctional constructs that combine biological tissues with engineered interfaces [25,29]. However, their incorporation requires careful consideration of material composition, dispersion, mechanical properties, and interactions with the surrounding biological environment.

Conductive fillers incorporated into hydrogel matrices represent a promising approach for modifying the electrical properties of biomaterials while preserving their suitability for three-dimensional tissue engineering. The available options are not equivalent, however, and their effects depend on filler geometry, concentration, dispersion, and interactions with the matrix. High-aspect-ratio carbon nanotubes can form conductive networks at relatively low loading, but comparisons with reduced graphene oxide and metallic nanoparticles depend on dispersion, surface chemistry, and matrix composition [25,59]. Conductive polymers such as poly(3,4-ethylenedioxythiophene) (PEDOT) offer an alternative, with hydrated conductivity and compatibility depending on formulation and processing [59].

Metallic nanostructures have also been used: hyaluronic acid-modified gold nanoparticles embedded in muscle bundles raised bulk conductivity and improved the locomotion of the resulting biohybrid robot, although the authors attribute the gain partly to enhanced myogenic differentiation rather than to conductivity alone, and no contractile force was measured [60]. Dose-dependent biological effects, the mechanical consequences of loading, and cost all require separate evaluation. A ranking of these filler classes cannot presently be supported. Doing so would require conductivity measured in the hydrated state at a stated loading and dispersion, reported alongside viable cell density, matrix stiffness, and stimulated force against a filler-free control prepared identically; no study compiled here reports that combination, and conductivity values are frequently absent even from dedicated comparisons of conductive biomaterials for muscle [59]. Conductivity gains alone do not establish improved excitation or force output.

The practical consequence is that filler selection involves a trade-off: enough conductive material must be present to form a continuous conductive path through the matrix, while the composite must remain compliant and printable enough to support myogenic differentiation [39]. Reported formulations balance these requirements differently, and no single filler is optimal across conductivity, dispersibility and biocompatibility simultaneously [59]. This constraint is one reason that electrode-level strategies, discussed in the following section, have so far advanced further than bulk conductive matrices as a route to uniform stimulation [25,29].

Conductive additives should therefore be evaluated through matched comparisons that report electrical properties, matrix mechanics, cell viability, and contractile output. This would help distinguish improvements associated with electrical stimulation from those arising from changes in tissue organization or matrix properties.

## 4. From Muscle Contraction to Robotic Function

**Figure 4:**
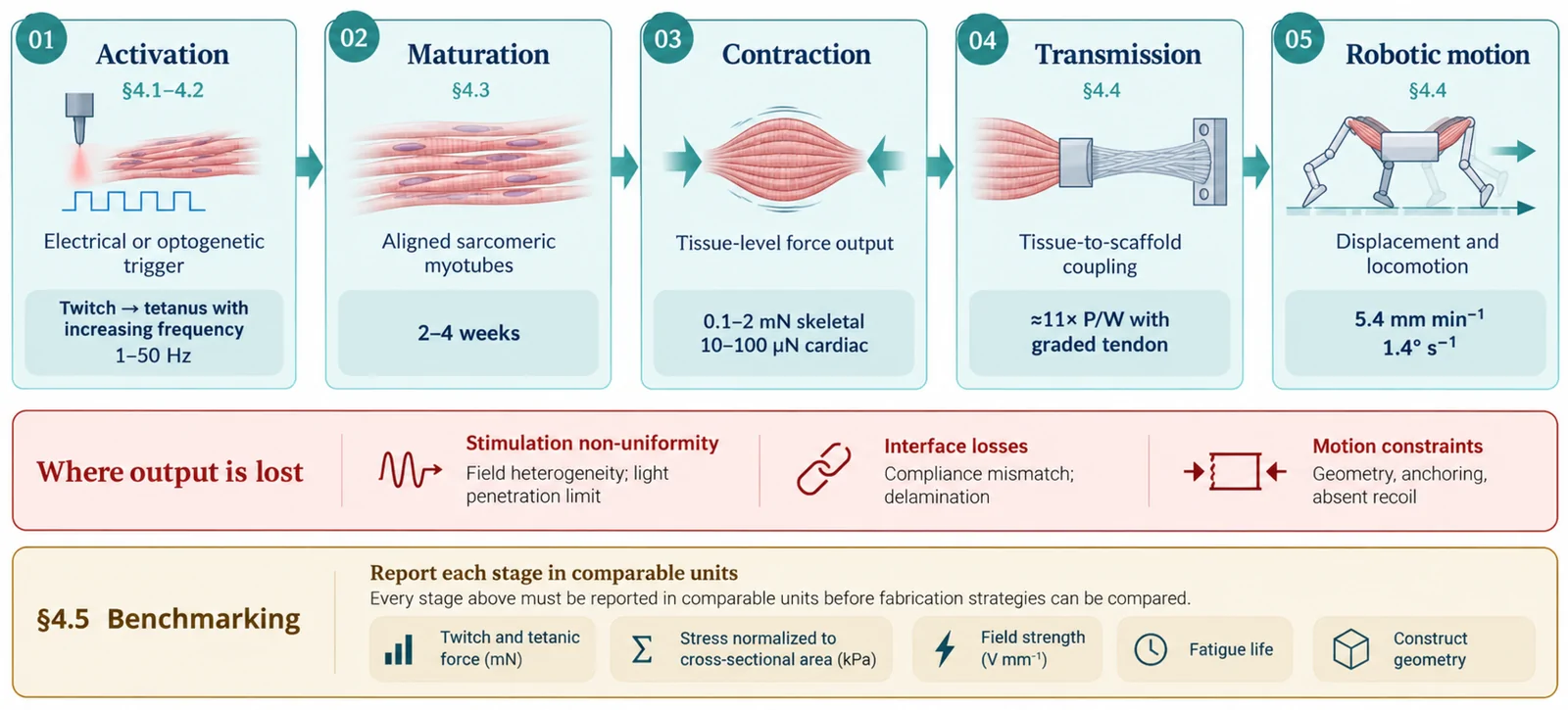
From contraction to robotic motion. Contractile output passes through five dependent stages, each constraining the next. Red band: the three points at which output is lost between stages. Amber band: the benchmarking parameters required before stages can be compared across studies (Section 4.5).

Generating a contraction and generating useful robotic motion are not the same problem. Between these two outcomes lies a chain of dependent processes: an activation signal must reach the tissue uniformly, the tissue must have matured enough to respond with adequate force, that force must be transmitted to a load-bearing structure with limited interfacial losses, and the resulting displacement must translate into a mechanically useful degree of freedom [4,29,33]. This section follows that chain, from electrical and optical activation, through mechanical conditioning and maturation, to force transmission and the benchmarking metrics needed to compare actuator performance across studies (Figure 4).

### 4.1. Electrical Stimulation

Unlike cardiomyocytes, which contract spontaneously through emergent syncytial electrophysiological coupling, engineered skeletal muscle can be activated using electrical stimulation or, following optogenetic modification, optical stimulation to initiate contraction [8,16]. This dependency is not merely a limitation; it is what enables skeletal muscle-based actuators to offer precise temporal control over the onset, duration, and magnitude of contraction, supporting programmable actuation [8,16]. Realizing this potential, however, requires integrated stimulation strategies that provide spatially controlled, sufficiently uniform activation throughout the engineered tissue, thereby introducing additional design constraints associated with actuator scaling and control [8,16].

Conductive materials embedded within the hydrogel matrix have emerged as one strategy for improving signal transmission and excitability. Conductive polymers such as PEDOT:PSS, carbon-based nanomaterials including carbon nanotubes and reduced graphene oxide, and metallic nanostructures have all been investigated as fillers for muscle-supporting matrices [59], with carbon nanotube–GelMA composites used directly in cardiomyocyte-driven soft robots [25] and carbon nanotube yarns in bioinspired artificial muscles [61]. Hyaluronic acid-modified gold nanoparticles embedded within muscle bundles have been reported to raise bulk electrical conductivity and to improve the locomotion of the resulting biohybrid robot, although the authors attribute the gain partly to enhanced myogenic differentiation rather than to conductivity alone [60]. At the electrode level, embedding pillar electrodes directly within the tissue rather than relying on external stimulation rods increased contractile distance from 13–82 µm with external gold rods to 108–165 µm at the same stimulus amplitudes, and it also increased the selectivity index between adjacent tissues from 0.33 to 0.94 [29]. Wireless optoelectronics provide a separate route to untethered stimulation of muscle-driven robots, as discussed in Section 4.2 [21].

Despite these advances, electrical stimulation remains constrained by field uniformity. External electrical fields often distribute nonuniformly across three-dimensional tissue constructs, producing regionally heterogeneous activation, asynchronous force production, and a net contractile output that falls short of what synchronous recruitment would achieve [32]. At high field intensities, local field strength near the electrode surface can cause membrane electroporation and direct cell damage without improving recruitment of cells deeper in the construct [32,62]. The conductive matrix additives and embedded pillar electrodes discussed above partially address field distribution by improving bulk electrical conductivity and concentrating stimulation within the tissue, but heterogeneous activation remains a limiting factor as construct dimensions increase [29,32]. Repeated or sustained stimulation can induce muscle fatigue, and progressively reduce force output; excessive stimulation or loading may also damage the tissue [4,32]. Stimulation protocols are also not standardized across laboratories, so voltage, frequency, and pulse geometry vary widely between studies, making comparison of reported force outputs difficult even when the underlying cell type and matrix are similar [4,32].

### 4.2. Optical and Optogenetic Stimulation

Optical stimulation offers an alternative activation route, generally through optogenetic modification of muscle cells, and provides an advantage over electrical stimulation by enabling activation without direct electrode contact, making it attractive for wireless, tether-free biohybrid designs [6,32]. Battery-free wireless optoelectronics have already been used to remotely control muscle-driven miniature robots, demonstrating this potential at the device level [21].

The trade-off is twofold. First, optogenetic activation requires expression of light-sensitive proteins such as channelrhodopsin-2 through genetic modification, adding a layer of biological engineering and regulatory complexity not present in electrically stimulated systems [6,32]. Second, the light has to reach the cells. Activation depth is set jointly by the illumination wavelength, the absorption and scattering properties of the construct, the irradiance delivered at its surface, and the sensitivity of the opsin, so no single penetration depth applies across systems; we are not aware of a study that reports activation depth as a function of these parameters for engineered muscle, and the values that would make such comparisons possible are among those listed in the reporting framework of Section 5.4. The optogenetic actuators discussed here express ChR2(H134R) [21,26], activated with blue light at 470 nm in the flexure-based system [26]. Where external illumination is insufficient, embedded sources offer a practical alternative: the battery-free wireless system of Kim et al. carries its optoelectronic light source on the device rather than illuminating the construct from outside [21]. For thicker constructs, such embedded sources or hybrid electrical–optical strategies are likely to be required in place of external illumination alone.

### 4.3. Mechanical Conditioning and Maturation

Before any stimulation strategy can produce a useful contraction, the tissue must first mature into a force-generating structure, and this maturation is itself governed by mechanical boundary conditions. Formation and functional maturation of engineered muscle can take days to weeks, depending on cell source, scaffold composition, and stimulation protocol [7,8]. Throughout this period, the construct remains mechanically vulnerable: repeated contraction cycles can cause detachment, collapse, or degradation of the supporting matrix if it is insufficiently robust [7,8]. Substrate stiffness and viscoelasticity, degradation kinetics, and the presentation of adhesion ligands together regulate myoblast alignment, fusion, and sarcomere organization, meaning that the mechanical environment during this window strongly influences the tissue’s eventual force-generating capacity [7,8,16,23].

Stable anchoring is what allows this maturation process to proceed without collapse. Defined boundary conditions and anchoring interfaces preserve tissue geometry as the myotubes mature, while simultaneously establishing the pathway through which contractile force will later be transmitted to the surrounding structure [19,21,26,29]. This dual role, structural support during maturation and force-transfer conduit during actuation, makes anchor design inseparable from stimulation and force transmission strategy. Custom PDMS ring molds, for instance, have been shown to act as a mechanical constraint that resists tearing during tissue compaction, thereby directly enhancing tetanus-driven contractile force output [19]. Tensegrity-based frameworks extend this principle by using selective contraction of multiple muscle tissues against a pre-tensioned structural network, converting a fixed anchoring geometry into a source of flexible, omnidirectional actuation [27].

### 4.4. Force Transmission, Recoil, and Degrees of Freedom

Once a mature, stimulable tissue exists, the dominant engineering bottleneck shifts from the biology of contraction to the mechanics of transmitting that contraction into robotic motion. The central challenge in scalable biohybrid actuation is not force generation in isolation, but the coordinated integration of biological contraction with a mechanically stable, stimulus-synchronized, load-bearing architecture [4,6,63]. Reported contractile forces from engineered muscle remain, when normalized by cross-sectional area, one to two orders of magnitude below those of native adult skeletal muscle [4,33], and this shortfall is commonly attributed to some combination of incomplete maturation, tissue architecture, normalization convention and losses at the tissue–material interface. Low specific force alone, however, does not distinguish between these contributions, and they are separable in principle. Maturation and the fraction of the cross-section that is actually contractile set the intrinsic ceiling: engineered constructs reach 0.72–4.6 kPa of active stress against 50–118 kPa in adult muscle [17,19], and shorten by roughly 1% of their length where native muscle shortens by 20% [26]. Recruitment, the proportion of fibers depolarized by a given stimulus, is seldom reported and cannot be recovered from endpoint force. Interface efficiency is the one term for which a direct measurement exists, and it does not support a dominant role: the engineered muscle–tendon junction reported in [20] withstands more than 5 mN while the tissue driving it generates 400 ± 200 µN, leaving an order of magnitude of unused transmission capacity. The intrinsic force deficit therefore appears to be dominated by maturation and active muscle fraction, while the transmission losses that do occur arise from mechanism compliance rather than from failure of the tissue–material junction itself, as the following paragraph sets out.

Native muscle transmits force through the myotendinous junction, a graded biological transition between compliant tissue and stiff tendon. Most current biohybrid designs instead present an abrupt transition between soft tissue and rigid synthetic backbone, and this mechanical mismatch creates stress concentrations, delamination, and reduced net force output even when the underlying tissue is highly functional [4,20]. Castro et al. [20] quantified the consequence of addressing this directly. Introducing a biohybrid tendon between engineered muscle and the skeleton, together with a fiftyfold stiffer transmission mechanism, raised the fraction of internally generated muscle force actually delivered to the skeleton from ∼1.3% to ∼37%, a ∼29-fold gain in transmission efficiency measured against the same group’s earlier compliant design with identical muscle. The same study reports a ∼11-fold higher power-to-weight ratio (109.6 nW mg^−1^ of muscle tissue) than previously published actuators built from the same C2C12 fibrin/Matrigel formulation. This second figure is a comparison across studies rather than a matched control, so it also absorbs differences in skeleton design, construct geometry and stimulation protocol, and it is normalized to muscle mass rather than to actuator or robot mass; the matched Fme/Fma comparison is the more direct evidence that interface and transmission design, not tissue output alone, set actuator-level performance. Bioprinting offers a route to reproducing such graded interfaces on demand, as demonstrated by multicellular muscle–tendon printing with spatially distinct matrix zones [18], but current methods remain low-throughput and rarely produce continuous material gradients, leaving this an open fabrication problem [32].

Force transmission alone is insufficient to produce useful motion; the actuator must be able to return to its resting configuration to generate cyclic or directional motion. Many biohybrid actuators produce motion in a single direction, a constraint that arises from several interacting factors rather than from absent recoil alone: actuator geometry, anchor placement, and the spatial distribution of the stimulation signal each limit the effective degrees of freedom. Single-direction deformation should be distinguished from true single-DOF behavior in a kinematic sense; a flat muscle strip anchored at both ends deforms in one axis regardless of whether a recoil element is present, and adding passive elasticity or an antagonistic tissue pair restores the resting configuration without expanding the range of accessible motion [27,63]. Swimmers and simple walkers built around a single actuating muscle unit typically operate within this constraint, producing repetitive but geometrically simple motion [21,28]. Tensegrity-based designs point toward a way past this limitation, showing that selective contraction of multiple tissues against a pre-stressed structural network can produce three-dimensional multi-DOF tilting rather than the single-axis deformation of a strip actuator [27]. Embedded pillar electrodes have similarly been shown to increase effective contractile displacement by improving spatial control over which regions of the tissue activate [29]. Taken together, these examples indicate that future actuators will need co-designed passive and active mechanical elements, in which engineered recoil structures, graded interfaces, and contractile tissue operate as a single integrated system rather than as separately optimized components [6,27].

### 4.5. Performance Metrics and Benchmarking

The preceding subsections describe a pathway, stimulation, maturation, and force transmission, whose output ultimately needs to be expressed in comparable, quantitative terms if fabrication strategies are to be evaluated against one another. At the construct level, confluent cardiomyocyte monolayers generate a peak twitch force of 10–100 µN, sufficient for endurance-based applications but modest relative to skeletal alternatives [7,8]. Engineered skeletal muscle constructs by contrast, have been reported to generate peak forces in the order of 0.1–2 mN, with reported active specific forces in the order of 1–10 kPa under tetanic stimulation [7,8]. Individual studies fall outside these bounds in both directions. Among the constructs compiled in Table 1, reported active stress ranges from 0.72 kPa [17] to 4.6 kPa [19], and tetanic force reaches 5.9 mN when four muscle rings are stacked in series [19]. That a compilation of thirteen representative studies yields only two directly comparable specific force values, spanning a sixfold range and straddling the lower bound quoted in the reviews above, is itself the clearest statement of the problem. These numbers, however, are only meaningful for cross-study comparison when reported alongside consistent geometric and stimulation parameters.

At present, this consistency is largely absent. Parameters such as twitch force, tetanic force, stress normalized to cross-sectional area, contraction frequency, fatigue lifetime, stimulation conditions, and construct geometry are reported inconsistently across studies, limiting quantitative comparisons between fabrication strategies and preventing reproducible optimization across laboratories [64]. This is not a cosmetic reporting issue: without a shared set of benchmarks connecting stimulation input, tissue-level force output, and actuator-level displacement or locomotion velocity, it remains difficult to determine whether a given improvement, in bioink composition, electrode design, or anchoring geometry, translates into better robotic performance or merely a more favorable measurement under non-comparable conditions [64]. Establishing standardized benchmarking protocols that link cellular force generation directly to system-level functional outcomes is therefore a necessary step for evaluating progress along the entire contraction-to-motion pathway described in this section. Four classes of ambiguity account for most of the present incomparability, and each is removable at no experimental cost. First, the contraction being reported must be identified: active force separated from passive tension, isometric from isotonic measurement, and twitch from tetanic stimulation, since these can differ by an order of magnitude in the same construct. Second, the state of the tissue at the moment of measurement must be given: temperature, days in culture at testing, and applied preload, all of which shift force output independently of any fabrication variable. Third, the statistical basis must be stated: the number of independent biological replicates as distinct from repeated measurements on a single construct, a dispersion measure, and the criterion used to declare mechanical failure or to exclude a construct. Fourth, device-level power must specify its normalization explicitly, both the quantity in the denominator—muscle tissue mass, actuator mass, or total system mass—and its dimension, since power per unit mass (W kg^−1^) and power per unit weight (W N^−1^) are not interchangeable and are both reported as “power-to-weight”. The value of 109.6 nW mg^−1^ reported in [20], for instance, is power per unit mass of muscle tissue alone, and is not comparable to a figure normalized to the assembled robot. These conditions complement rather than replace the minimum reporting dataset proposed in Section 5.4, which specifies what should accompany a published measurement; the items here specify the conditions under which it was made.

## 5. Barriers to Scalable and Autonomous Biohybrid Robots

Although biohybrid actuators have shown significant promise, numerous aspects remain unexplored and several technical challenges persist [5]. Rather than arising from a single material or biological limitation, these challenges stem from tightly coupled interactions among tissue architecture, force-transfer interfaces, nutrient transport, stimulation uniformity, manufacturing constraints, and control strategies, all of which become increasingly interdependent as actuator dimensions scale [4,6,63]. Here, we discuss the key obstacles toward achieving consistent force generation and outline a roadmap for the next generation of biohybrid robotics. These challenges can be considered across multiple levels of integration, ranging from tissue maturation and biological functionality to actuator design, robotic integration, manufacturing, and regulatory considerations [4,6].

### 5.1. Tissue-Level Challenges

As biohybrid actuators increase in size and structural complexity, maintaining uniform tissue viability, maturation, and reproducible functionality becomes increasingly challenging due to limitations in transport, biological variability, and construct heterogeneity [4,5,65]. Engineered muscle tissues require tightly regulated physiological conditions, including appropriate medium composition, glucose availability, and pH, to maintain cellular activity and support long-term function in vitro [5,33]. Therefore, scaling biohybrid systems beyond small tissue dimensions requires strategies that ensure sufficient oxygen and nutrient delivery while maintaining a suitable microenvironment for tissue maturation [4,5].

While diffusion can support cellular survival in relatively thin constructs, it becomes increasingly limiting as tissue dimensions increase, creating gradients in oxygen concentration, nutrient availability, and metabolic waste accumulation [65]. To overcome these limitations, approaches such as perfusable networks and sacrificial-material-based microchannels have been explored to improve mass transport within large engineered tissues [65]. Although multi-material 3D printing provides opportunities to integrate complex perfusable architectures into biohybrid actuators, maintaining fabrication precision, throughput, and reproducibility remains a significant challenge [10,47]. Thus, transport engineering represents not only a strategy for preserving tissue viability but also a key requirement for achieving spatially uniform maturation and reproducible actuator performance at larger scales [4,5].

Beyond transport limitations, biological variability remains a major obstacle for the reproducible manufacturing of biohybrid actuators. Differences in cell differentiation, maturation state, and tissue organization can lead to substantial variation among nominally identical constructs, reducing predictability and complicating systematic optimization [4,33]. These variations become increasingly significant as systems scale from simplified tissue models to larger, more complex robotic platforms, where small differences at the cellular level can translate into measurable differences in overall device performance [33,65].

Collectively, these challenges demonstrate that scaling biohybrid actuators requires simultaneous control over biological development, mass transport, and manufacturing reproducibility. Addressing these factors will be essential for transitioning biohybrid systems from individually optimized laboratory demonstrations toward reliable and scalable platforms.

### 5.2. Interface- and Actuator-Level Challenges

Beyond biological tissue development, biohybrid systems face broader challenges associated with integrating living components into engineered robotic platforms. Unlike conventional engineering approaches, which typically optimize synthetic materials for predefined functions, biohybrid design requires coordinating biological and artificial components with fundamentally different properties, response times, and failure mechanisms [63]. This creates unique challenges in controlling interactions between living tissues and synthetic structures while maintaining reliable long-term operation.

Although advances in tissue engineering and biofabrication have improved the complexity of engineered muscle constructs, current biohybrid actuators remain limited by reduced scalability, manufacturing complexity, and incomplete understanding of biological–synthetic integration compared with fully synthetic robotic systems [4,6]. Increasing construct size, cell density, and architectural complexity can improve biological functionality, but these approaches also introduce additional challenges in fabrication accuracy, quality control, and reproducibility [33].

A further challenge is that biohybrid systems require integrating biological materials with artificial components that may differ substantially in mechanical properties, degradation behavior, and environmental requirements. Developing reliable manufacturing strategies, therefore, requires not only advances in biomaterials and fabrication technologies but also new approaches for coordinating biological variability with engineering constraints [63].

From a translational perspective, the fabrication of biohybrid robots remains limited by the difficulty of producing standardized, scalable, and reproducible systems. Current approaches often rely on highly customized experimental designs optimized for individual demonstrations, making comparison between studies and systematic improvement challenging [6,63]. Future progress will therefore depend on manufacturing strategies that enable consistent production, integrated quality assessment, and predictable performance across larger device populations.

### 5.3. System-Level Challenges

From a technical perspective, scaling biohybrid actuators from small prototypes to larger, practical devices remains a significant challenge [6,63,65]. Practical issues, such as large-scale production, transportation, and storage, also remain unresolved [63]. The integration of engineered muscle tissue complicates matters further, as many muscle constructs operate at millimeter scales, while external support hardware can dominate total system mass and volume [65]. Addressing these challenges requires multidisciplinary efforts to develop scalable fabrication techniques that enable the achievement of functional muscle tissues, seamless bio-synthetic integration, and robust control. Overcoming these barriers is essential for realizing autonomous, macro-scale biohybrid soft robots for real-world applications [6,63,65,66]. The practical deployment of biohybrid actuators is further constrained by cost, storage, and operational lifespan, each of which represents a bottleneck that laboratory demonstrations do not consistently resolve. Fabrication costs are driven primarily by cell culture consumables, specialized biomaterials, and the skilled labor required for tissue formation and quality control; unlike conventional robotic components, biological actuators are not yet routinely manufactured and stored as off-the-shelf parts [6,63]. Reported operational lifespans are hard to compare because the published figures measure different quantities. The battery-free walking robot of Kim et al. remained functional for two to three weeks with aminocaproic acid supplementation, with force and locomotion characterised on days 15 to 21 [21], whereas bioprinted muscle–tendon units retained contractile function over a three-month culture period [18]. Neither figure describes continuous operation: both refer to constructs held in culture and actuated intermittently during testing. Where contractility is tracked across the culture period, it also passes through a maximum, peaking at day 10 in tensegrity actuators [27] and day 12 in pillar-electrode constructs [29] before declining, so the interval over which an actuator performs usefully is shorter than the interval over which its tissue survives. Comparable lifetimes would require a stated failure criterion—loss of viability, decline of force below a defined fraction of peak, or inability to complete the actuator’s task—together with whether the device was operated continuously or tested intermittently with culture maintenance between measurements. The underlying limits are tissue degradation under mechanical loading, accumulation of metabolic waste, and the difficulty of exchanging culture media inside a sealed or operating robotic system [6].

Beyond fabrication challenges, the transition from proof-of-concept bioactuators to autonomous biohybrid robotic systems requires predictive design frameworks that integrate biological variability, environmental feedback, and adaptive control [6]. One of the key challenges facing all classes of biohybrid robotics is the lack of unified or broadly adopted design and modeling tools. For tissue-engineered robots, design frameworks must capture and predict the complex, often stochastic nature of biological tissue formation. The biological components underlying these robotic platforms exhibit many unresolved aspects in their communication systems, including interconnectivity pathways and the mechanisms by which biological feedback loops generate control. Additionally, predictive models are needed to determine how control signals influence behavioral responses. Design tools must also account for the effects of integrating microelectronic systems with living tissues, including changes in load distribution and proprioceptive feedback. For aquatic systems in particular, considerations such as buoyancy and added mass in variable-density environments further complicate the design process [6].

Beyond stimulation, robust control of biohybrid actuators is still limited by natural biological variability and the lack of intelligent environmental perception [6]. Open-loop control systems, which function without feedback, are inherently restricted in their ability to adapt to unpredictable environments. Consequently, the implementation of closed-loop control systems has become increasingly important and requires integrating biological or artificial sensors that provide real-time feedback and enable autonomous responses [6]. However, this integration introduces additional fabrication and reliability challenges, particularly in maintaining stable and reproducible inputs and outputs over extended periods [6]. Advances in modeling, artificial intelligence (AI), and machine learning are therefore expected to contribute to the development of autonomous control strategies in biohybrid robots. Nevertheless, ensuring repeatability and robustness remains an unresolved challenge shared with broader fields, such as soft robotics [6].

### 5.4. Manufacturing and Standardization Challenges

Achieving three-dimensional muscle architecture using bioinspired materials is a technically challenging process that involves fabricating complex, anisotropic, multi-material structures [63]. Combinations of functional materials with a wide range of chemical and mechanical properties, and that feature details from the nanoscale up to the macroscale, demand advanced manufacturing tools and optimized methods that are reliable, fast, and cost-effective [63].

Despite advances in fabrication strategies, practical manufacturing challenges remain unresolved. Current approaches require further development toward scalable fabrication techniques that can produce functional muscle tissues, enable seamless biosynthetic integration, and improve reproducibility across platforms [6,63,65,66].

Maintaining sterility throughout fabrication is a practical challenge that is rarely addressed explicitly in biohybrid actuator studies, despite being a prerequisite for tissue viability and long-term operation. Conventional sterilization methods are incompatible with one or multiple components of these systems: autoclaving damages thermolabile hydrogels and polymers; gamma irradiation alters the mechanical and degradation properties of collagen and GelMA-based scaffolds; and ethylene oxide, while widely used for polymer sterilization, requires extended degassing periods and may affect bioactive surface coatings [67,68]. Electronic and mechanical components add a further constraint, as they cannot undergo the same sterilization treatments as biological scaffolds without risk of damage to circuits or insulation. No single protocol covers these architectures, and the procedures that are reported are specific to the device. In the tensegrity actuator of Morita et al., the printed anchors and PDMS culture devices were sterilized with ethanol and ultraviolet irradiation before cell seeding [27]; in the pillar-electrode system of Li et al., the electrodes and culture device were instead treated with 30 min of ultraviolet irradiation and ozone [29]. Both confine sterilization to the synthetic components and perform every subsequent step involving living tissue under aseptic conditions, but the method follows from the materials present, and neither protocol transfers unchanged to the other device.

Encapsulation is a barrier rather than evidence of sterility, and the tests that accompany it assess material compatibility rather than validating the assembled device. The pillar-electrode study illustrates what such testing comprises: parylene-C conformal coatings of 2 and 10 µm on the printed anchor and the wire joint, respectively; extract cytotoxicity of the exposed alloy electrode assessed against ISO 10993-12 at a contact ratio of 3 cm^2^ mL^−1^ over one, three and five days; and direct-contact viability measured on 3D muscle tissue after six days [29]. Each test addresses one surface—coating, electrical joint, cell-contacting electrode—and none establishes that the completed assembly can be rendered sterile and kept so. However, as biohybrid assemblies increase in complexity, incorporating wireless stimulators, embedded sensors, or multi-material junctions, the risk of contamination at interfaces that are difficult to access after assembly increases accordingly. Standardized sterile assembly protocols validated for specific biohybrid architectures have not yet been established, and this gap represents a practical barrier to moving beyond single-use laboratory prototypes.

A further limitation is the absence of standardized benchmarking metrics across studies, as described in Section 4.5. Both reporting differences and measurement variability contribute to this problem, so community agreement should address protocols, calibration, and data interpretation. Table 4 summarizes the principal manufacturing and standardization challenges, their functional consequences, and the strategies currently being pursued to address them.

Comparable problems in other biological fields have been addressed through minimum-information reporting standards, which define the smallest set of parameters that must accompany a published measurement for it to be independently evaluated and reproduced [69]. We propose an equivalent three-part framework for biohybrid actuators, covering reporting, sharing, and validation.

*Reporting.* A minimum dataset should accompany every reported actuator: (i) cell source, species, passage number, and differentiation duration; (ii) matrix composition and final concentration; (iii) construct geometry, specifically length, cross-sectional area, and anchor-to-anchor spacing; (iv) for electrically stimulated constructs, the applied voltage or current, the electrode material, dimensions and separation, the conductivity of the culture medium, and the nominal field strength in V mm^−1^ obtained from voltage and electrode separation, reported as a geometric estimate for that electrode configuration and medium, not as a measurement of the field within the tissue, which is neither uniform nor directly accessible; (v) for optically stimulated constructs, the opsin variant and its delivery method, the illumination wavelength, the irradiance at the tissue surface, the pulse duration and duty cycle, and the position and geometry of the light source relative to the construct; (vi) force as absolute twitch values in mN and as stress normalised to cross-sectional area in kPa, with the measurement method, the area used for normalisation, whether passive force was subtracted, and the loading condition (isometric or isotonic) all stated, together with tetanic force for skeletal muscle, an endpoint that does not apply to cardiac constructs, whose long refractory period prevents fusion into tetanus and for which spontaneous beating rate and rate control are the appropriate measures; (vii) fatigue expressed as cycles to a defined percentage of force decay under a stated protocol; and (viii) for assembled devices, displacement, locomotion velocity normalised to body length, and power-to-mass ratio with the mass basis defined. Three of these warrant emphasis because their omission is what most often makes studies incomparable: voltage alone does not define the field experienced by cells, force alone does not establish intrinsic tissue performance, and an endpoint appropriate to one muscle phenotype may be meaningless for the other.

*Sharing.* Raw force traces, construct dimensions, and stimulation logs should be deposited in a general-purpose public repository with a persistent identifier, in machine-readable form rather than as figures. This is presently rare, and the resulting scarcity of structured data prevents both independent reanalysis and the assembly of training sets large enough to support the data-driven design approaches discussed in Section 6.1 [70].

*Validation.* Reporting and sharing establish comparability only if the underlying measurements are calibrated. We suggest the field adopt a reference construct of defined cell source, matrix, and geometry, which laboratories can fabricate and measure to characterise their own force-measurement setup alongside novel designs. Inter-laboratory comparison of such a reference would quantify the between-laboratory variability that is at present difficult to separate from biological variability, and would establish whether differences between published actuators reflect genuine design improvements or differences in measurement practice.

This framework builds on established reporting and data-management practices, but inter-laboratory validation will require coordination, resources, and agreement on reference protocols. Standardization should therefore develop alongside biological and engineering advances, as discussed in Section 5.6.

**Table 4:** Key manufacturing and standardization challenges in muscle-based biohybrid actuators, with current limitations and emerging mitigation strategies.

Challenge	Core Bottleneck	Impact on Actuator Performance	Current Approaches	Emerging Mitigation Strategies	Refs
Biological variability	Batch-to-batch differences in primary cell isolation and inconsistent differentiation efficiency lead to variable muscle progenitor quality across experiments	Unpredictable contractile force output and maturation timing; limits reproducibility and cross-laboratory comparison	Immortalized cell lines (C2C12); standardized differentiation protocols; viability and morphology quality-control assays	iPSC-derived myogenic cells from defined genetic backgrounds; automated culture workflows; AI-based quality screening of bioink formulations	[6,13,64]
Absence of benchmarking standards	No consensus on which performance metrics to report (twitch force, tetanic force, specific stress, fatigue lifetime, stimulation conditions, construct geometry); normalization strategies differ widely across studies	Quantitative comparison across studies and laboratories is impossible; reproducible optimization is prevented; fabrication strategies cannot be ranked objectively	Ad hoc reporting; some groups normalize force to cross-sectional area; inconsistent stimulation protocols	Community-wide minimum reporting standards; open access benchmarking datasets; standardized stimulation and measurement protocols	[7,64,70]
Scalable fabrication	Constructs are limited to millimeter-scale; the issue of increasing size while preserving anisotropic cellular organization, mechanical integrity, and cell viability remains unsolved	Force output does not scale proportionally with construct volume; functional complexity is constrained to simple geometries	Modular assembly of smaller tissue units; mold-based casting; ring and pillar formats that maximize surface-to-volume ratio	Multimaterial bioprinting with integrated transport networks; modular biofabrication; hierarchical scaffold architectures	[63,66,71]
Nutrient and oxygen transport	Passive diffusion limits viable tissue thickness to approximately 200 µm; larger constructs develop necrotic cores without active perfusion	Functional actuator cross-section is constrained; force generation per unit volume decreases as construct size increases	Thin-film and ring geometries; co-axial extrusion to create hollow channels; sacrificial templating for internal voids	Engineered perfusable microvascular networks; oxygen-releasing biomaterials; bottom-up modular assembly with integrated microfluidics	[72,73,74,75]
Data interoperability for AI	Published datasets use incompatible formats and metadata, and public deposition of raw data remains uncommon, limiting model generalizability	Machine learning models trained on narrow datasets fail to generalize across laboratories, bioinks, or cell sources; slows development of predictive design tools	Lab-internal databases; limited open repositories; manual extraction of parameters from publications	Standardized data ontologies for biohybrid actuators; federated learning across laboratories; multimodal datasets linking fabrication parameters to functional outcomes	[64,70,76]
Long-term viability and fatigue	Engineered muscle tissues degrade under sustained mechanical and electrical loading; metabolic demand increases with maturation; media exchange inside closed robotic systems is impractical	Operational lifespan currently measured in days to weeks; limits deployment in autonomous or real-world applications	Controlled stimulation protocols with rest periods; biomaterial scaffolds with tunable crosslinking to buffer mechanical stress	Self-healing and dynamically remodeling hydrogels; closed-loop fatigue monitoring; metabolic self-sustaining strategies	[6,77,78,79]

Despite growing interest in AI-driven approaches, the primary limitation to expanding AI applications in biohybrid actuators is not a lack of algorithms but the scarcity of standardized, high-quality datasets [64,70]. Most studies report fabrication parameters and biological outcomes in formats that are difficult to analyze computationally, limiting interoperability between studies [70]. As a result, AI models trained on narrow or laboratory-specific datasets may achieve strong internal performance yet fail to generalize across laboratories, printers, bioinks, or cell sources [76]. Accordingly, a major barrier to AI-enabled biohybrid actuator design is the absence of interoperable, actuator-specific datasets that link fabrication parameters to functional robotic outputs. The scale of the shortfall is visible in the only direct example available: the published machine-learning study of muscle biohybrid actuators discussed in Section 6.1 draws on 161 experiments from a single actuator configuration. Reviews of machine learning in biofabrication reach a comparable conclusion for bioprinting, where model performance is constrained by the amount and type of data available rather than by the choice of algorithm [70,76]. Public deposition of raw force traces, construct dimensions and stimulation logs remains uncommon in this literature, although we are not aware of a survey that quantifies the rate; the reporting and data-sharing framework proposed in Section 5.4 addresses that gap directly and is therefore a precondition for AI-assisted biohybrid robotics rather than merely good practice. In the long term, integrating AI models with real-time sensing systems, mechanistic simulations, and actuator-level performance data could enable the development of digital twins and adaptive closed-loop design frameworks for biohybrid robots [80,81]. However, such systems will continue to rely heavily on experimental validation, as biological maturation and contractile functionality cannot yet be reliably predicted solely from fabrication parameters [82,83].

### 5.5. Ethical and Regulatory Considerations

Ethical considerations in biohybrid robotics extend beyond conventional tissue-engineering concerns because these systems integrate living contractile tissues into autonomous or semi-autonomous engineered platforms [8,64]. These considerations become increasingly important as biohybrid systems move beyond controlled laboratory environments toward more integrated and potentially deployable robotic platforms [64,84].

The development of bioactuators based on engineered skeletal and cardiac muscle raises several ethical concerns spanning biological sourcing, animal welfare, biosafety, and broader social implications [64,85]. The primary ethical issue relates to the origin of the muscle cells. When derived from animal tissue, researchers must ensure compliance with the principles of Replacement, Reduction, and Refinement to minimize animal suffering and the number of donor animals used [4,85]. For human-derived cells, issues of informed consent, donor privacy, and ownership of biological materials become central [30]. Moreover, common culture supplements such as fetal bovine serum raise additional ethical and sustainability questions due to their animal origin, prompting recent calls for the adoption of xeno-free culture systems [86].

Furthermore, societal and environmental responsibilities are critical considerations. Researchers must ensure the safe containment, use, and disposal of hybrid biological materials to avoid ecological harm [85,86]. Equitable access, fair distribution of benefits, and transparency regarding the purposes and potential applications of these systems are highlighted throughout the literature [4,86]. Therefore, ethical responsibility in biohybrid actuators extends beyond laboratory compliance; it requires active consideration of sourcing ethics, biosafety, and the social implications of merging biological and robotic domains [64,86].

Another important aspect concerns biosafety and the handling of genetically modified cells [64,86]. Optogenetic control of biohybrid actuators requires expression of light-sensitive proteins such as channelrhodopsin in muscle cells, introduced by transgenic modification [65,66], as in the optogenetic C2C12 constructs used for wireless walking robots and flexure-based actuators [21,26]. Where a viral vector is used, containment measures should be selected through an institutional risk assessment that considers the vector, transgene, host cells, procedures, and the applicable national and institutional requirements, which differ between jurisdictions [87]. Once cells have been stably transduced and residual vector has been cleared, containment requirements may be reduced, but this determination must follow the same formal risk assessment rather than be assumed [87]. A specific challenge for biohybrid actuators is that genetically modified tissue is integrated into a mechanical device, which complicates both containment and disposal: the assembled construct may contain electronic and polymer components that are incompatible with autoclaving, so decontamination protocols must address biological and synthetic materials simultaneously. Whether optogenetic responsiveness remains stable over the operating life of a construct is also rarely addressed: performance is typically characterised at a single stage of maturation rather than tracked across the culture period, so any drift in opsin expression or light sensitivity would not be visible in the data as currently reported. Studies using genetically modified cells in biohybrid actuators should therefore explicitly report the vector system, the stability of transgene expression across the reported culture period, biosafety classification, institutional approval obtained, and disposal protocol followed [64,86]. More broadly, as biohybrid systems increasingly blur the boundary between living and artificial entities, explicit ethical reflection, transparency, and public engagement remain essential [64,86]. When neural integration is introduced for actuation or feedback, questions arise regarding potential sentience or nociceptive capacity, although non-innervated muscle tissues are generally considered unlikely to raise comparable welfare concerns [86].

### 5.6. Priority Ranking of Challenges

The challenges described in this section are coupled: biological maturation, force transmission, stimulation control, and scalable manufacturing constrain one another rather than acting independently, so gains in one component do not necessarily improve overall performance [63,64]. The ordering below is an author proposal rather than a validated ranking, and it applies three criteria: the expected gain in functional scale if the constraint were removed, the expected gain in reproducibility across constructs and laboratories, and the tractability of a solution using methods now available. Weighting these differently changes the order. For an in vitro drug-screening platform, where constructs stay small and are never required to locomote, reproducibility dominates and nutrient transport falls well down the list; for an untethered centimetre-scale walker, transport and operational lifespan become the binding constraints; and for any device intended for implantation or environmental release, biosafety and regulatory readiness govern what can be attempted at all, irrespective of technical maturity. The list below therefore separates technical bottlenecks, which are ranked, from requirements that run alongside them and cannot be deferred.

**Figure 5:**
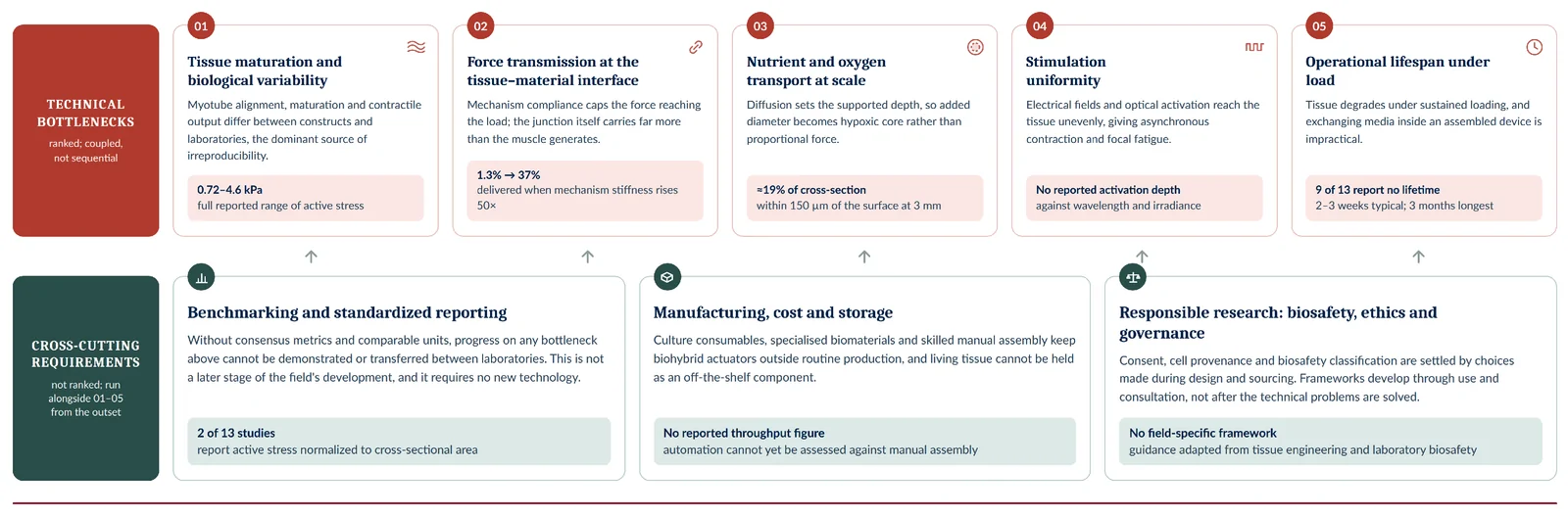
Proposed priorities for near-term progress in biohybrid actuators. Items 01–05 are technical bottlenecks in the contraction-to-motion pathway, ordered as in Section 5.6; they are coupled rather than sequential, and each constrains the others. The three cross-cutting requirements below are deliberately unnumbered: they are not a later stage that follows the technical work but run alongside it from the outset, as the upward arrows indicate. Benchmarking determines whether progress on a bottleneck can be demonstrated at all, manufacturing constraints shape how each solution must be built, and questions of consent, provenance and biosafety are settled by choices made during design and cell sourcing. Values shown are drawn from the studies compiled in Table 1 and from Section 4.5; the ordering reflects the authors’ engineering assessment rather than a validated ranking.

Proposed priorities for achieving functional, scalable biohybrid actuators (Figure 5) include the following:

1. **Tissue maturation and biological variability:** Inconsistent myotube alignment, maturation, and contractile output remain important sources of irreproducibility across laboratories. Incorporating motor neurons and functional neuromuscular junctions could support muscle maturation while shifting skeletal muscle actuation from direct external stimulation toward more physiologically relevant, biologically mediated control. Establishing a well-characterised and reproducible biological baseline, potentially encompassing such neuromuscular complexity, is therefore essential to reliably assess downstream engineering improvements.
2. **Force transmission at the tissue–material interface:** Compliance mismatches and poor adhesion between living tissue and structural scaffolds impose a ceiling on how much contractile force reaches the load, regardless of biological quality. Its contribution should be measured separately from tissue-level force generation.
3. **Nutrient and oxygen transport at scale:** Diffusion limits viable tissue thickness to the submillimeter range, constraining the viable muscle volume available for force generation. Perfusion, vascularization, or architectures that shorten diffusion distances may help overcome this limitation.
4. **Stimulation uniformity:** Spatially heterogeneous electrical or optogenetic activation produces uneven contraction and accelerated focal fatigue. Achieving uniform excitation across millimeter-to-centimeter constructs remains an open engineering problem.
5. **Operational lifespan under load:** Engineered muscle degrades under sustained mechanical and electrical loading, and maintaining it inside an assembled device is impractical with current approaches, confining reported operation to timescales of weeks.

Three further requirements are not stages that follow the list above. They run alongside it, and progress on them is neither gated by nor a substitute for progress on the technical bottlenecks:

- **Benchmarking and standardized reporting:** Without consensus metrics for contractile force, strain, fatigue life, and power density, cross-laboratory comparison is difficult and field-wide progress cannot be tracked. Standardization is not a later stage of the field’s development but the mechanism by which progress on items 1–5 becomes visible and transferable, and it requires no new technology (Section 5.4).
- **Manufacturing, cost, and storage:** Reliance on cell-culture consumables, specialized biomaterials, and skilled manual assembly keeps biohybrid actuators outside routine production, and living tissue cannot be held as an off-the-shelf component. These constraints restrict deployment to controlled laboratory settings independently of how the technical bottlenecks are resolved.
- **Responsible research—biosafety, ethics, and regulatory engagement:** Frameworks for biohybrid systems remain nascent, but they develop through use and consultation rather than after the technical problems are solved. Deferring them until devices are sufficiently functional would leave the field without governance experience at the point where translation becomes possible.

Items 1–3 are the most tightly coupled: progress on biological variability, interfacial mechanics, and nutrient transport is a precondition for the functional scale that the remaining technical items presuppose. This does not place benchmarking or responsible research behind them. Concentrated effort on those three, pursued alongside the cross-cutting requirements, is, for our assessment, the highest-leverage path toward functional, deployable biohybrid actuators.

## 6. Future Perspectives and Emerging Trends

The future development of biohybrid robots will depend on the transition from isolated proof-of-concept demonstrations toward predictable, manufacturable, and autonomous actuator systems [6,64]. Although recent advances in tissue engineering, biofabrication, computational modeling, and biomaterials have significantly expanded the capabilities of muscle-powered biohybrid robots, major challenges remain in controlling biological variability, scaling fabrication processes, and achieving reliable long-term operation [13,71]. Rather than introducing technologies in isolation, future progress will require integrating data-driven design, scalable biofabrication strategies, adaptive materials, and intelligent control systems [6,64,71]. In this context, three capabilities are expected to define the next generation of biohybrid actuators: predictable design, manufacturable fabrication, and autonomous operation.

### 6.1. Predictable Actuators: AI, Digital Twins, and Data-Driven Design

One of the major limitations in current biohybrid actuator development is the predominantly empirical nature of design optimization, where fabrication parameters and biological outcomes remain difficult to predict across different systems [64,76]. Unlike conventional robotic systems, in which mechanical properties and performance can often be predicted using well-established engineering principles, biological components exhibit substantial variability due to differences in cell behavior, maturation, remodeling, and degradation [4,6]. Future biohybrid actuators will require predictive frameworks that connect fabrication parameters, biological development, and actuator-level performance through approaches such as data-driven modeling and digital twins [6,80]. The following discussion distinguishes predictive modelling of actuator output, learning-based control, inverse design and parameter optimization, digital twins, and automated image-based characterization; closed-loop control at the device level is addressed in Section 6.3.

Direct AI and machine learning applications to muscle-based biohybrid actuators remain limited, but the first examples have now appeared. In a preprint, Tsompanas et al. applied supervised learning to experimentally characterized biohybrid actuators consisting of skeletal muscle rings anchored on flexible polymer pillars [88]. From 161 experiments performed on a single actuator configuration, random forest and neural network regressors predicted the peak force of each experiment from the muscle-ring sample number together with stimulation frequency, pulse width and waveform, reaching $R^{2}$ of 0.93 and 0.94, the latter after adding unstimulated baseline force as a further input; a long short-term memory network trained on overlapping ten-step windows of measured force forecast the next force value with $R^{2}$ of 0.9956 [88]. Two features of the evaluation bound what these figures establish. The static models were assessed on a random 80/20 split of individual experiments rather than a split by actuator, and ring identity was itself a model input, so the reported accuracy describes interpolation within already-characterized actuators rather than prediction for a new one. The dynamic model was trained and tested on 122,176 overlapping windows drawn from the same recordings, so neighbouring training and test windows share nine of their ten samples. Demonstrating that such models generalize would require held-out-actuator validation, in which every measurement from a given construct falls on one side of the split.

Separately, Schaffer et al. developed a reinforcement learning framework to coordinate 42 simulated muscle units in a lattice-structured biohybrid robot, showing that agents trained with models of exercise-induced muscle adaptation outperformed those using static muscle models [89]. That work operates entirely in simulation, but it demonstrates that reinforcement learning can exploit biological plasticity as a design variable rather than treating variability as noise. Together, these studies show that data-driven methods can capture aspects of biohybrid actuator behavior, while also showing how far the evaluation protocols are from what general design workflows would require.

In conventional robotics and biofabrication, AI has already demonstrated significant potential in structural optimization, environmental perception, fabrication parameter optimization, and the analysis of biological outcomes [76,90,91,92]. Although most current AI applications in this space have focused on biofabrication processes rather than complete biohybrid robots, these approaches provide a foundation for developing actuator-specific predictive models by linking fabrication parameters with biological and functional outcomes [64,76,91].

Future AI-driven design frameworks could integrate multiple parameters simultaneously, including scaffold geometry, material composition, mechanical properties, cell density, tissue alignment, stimulation conditions, and actuator architecture [64,80]. Rather than predicting individual fabrication outcomes, inverse design approaches could identify combinations of biological and mechanical parameters required to achieve target functions such as force generation, bending, gripping, locomotion, or long-term stability [93,94]. Recent studies in scaffold and implant design demonstrate how surrogate models and optimization algorithms can connect desired mechanical or biological properties with printable architectures, providing a conceptual framework for future biohybrid actuator design [95,96]. Applying these strategies to muscle-powered actuators will require optimization criteria that account not only for static mechanical properties but also for dynamic parameters such as contractile force, fatigue resistance, and biological maturation [4,33,64].

Digital twins represent a longer-term objective for biohybrid actuator design. The LSTM framework of Tsompanas et al. is presented by its authors as a digital twin [88], and it does reproduce a measured force trace closely, but what it demonstrates is one-step-ahead forecasting within a fixed dataset rather than a model that tracks a living construct as it matures, adapts, or fatigues. The distinction is worth keeping, because the two have different requirements. A fitted predictor needs a representative training set; a digital twin additionally requires state measurements taken from the construct during operation, an update frequency matched to the timescale of the biological change being tracked, calibrated uncertainty on its predictions, and validation against constructs that contributed no training data. Beyond these, a full biohybrid twin would have to represent both the synthetic structure and the living tissue, and to track how geometry, material properties, culture conditions, and stimulation history jointly shape performance over time [6,80], none of which conventional mechanical simulation is built to do [4,6]. Until those elements are specified and tested, the term names an objective rather than a demonstrated capability.

AI-based monitoring and characterization will further support the development of predictable actuators [64]. Machine learning approaches have demonstrated the ability to extract functional information from complex biological datasets, including contractility measurements, microscopy images, and fabrication outputs [83,97,98]. Extending these approaches to biohybrid actuators could enable automated evaluation of parameters such as contraction amplitude, force generation, tissue organization, and fatigue behavior [6]. These approaches depend directly on the reporting and sharing practices set out in Section 5.4; in their absence, AI-driven optimization will remain limited by poor generalizability [64,70].

Together, these approaches could transform biohybrid actuator development from iterative trial-and-error optimization to predictive design workflows, in which biological and mechanical parameters are jointly optimized before fabrication [64,80,93].

### 6.2. Manufacturable Actuators: Scalable Biofabrication and Multi-Scale Integration

Predictable design alone will not be sufficient to advance biohybrid robotics; future actuators must also be manufactured with high reproducibility, structural complexity, and scalability [63,71]. Achieving functional muscle-based actuators requires fabrication strategies that simultaneously control cellular organization, mechanical properties, and integration with robotic architectures. Advanced biofabrication approaches, including multimaterial 3D printing, modular assembly, and engineered biomaterials, are therefore expected to play a central role in overcoming current manufacturing limitations and enabling the transition from laboratory-scale constructs toward practical biohybrid systems [63,71].

One future direction is the use of stimuli-responsive matrices that actively shape tissue organization rather than passively supporting it [99,100,101]. Two capabilities relevant to muscle actuators have been demonstrated at the materials level: the stiffness of cell-laden protein–polymer hydrogels can be modulated reversibly in response to external triggers including light [102], and printed composites can be programmed to change shape after fabrication through anisotropic swelling [103]. Both point toward matrices that impose mechanical cues during maturation rather than fixing them at the moment of casting, though neither has yet been applied to a contractile muscle construct. Computational methods have in parallel accelerated bioink development, predicting gelation, viscosity and printability from formulation and process parameters rather than establishing them empirically [92,104,105,106]. Self-assembly offers a complementary route to architecture: cells printed into defined topologies reorganise into coherent tissue without an imposed scaffold [107,108], and in skeletal muscle specifically, anisotropic alignment has been achieved through co-axial extrusion into fascicle-like bundles [41], embedded guidance fibres [42], and magnetically oriented nanofibre scaffolds [109]. What remains unreported is the step that matters most for actuators: no stimuli-responsive matrix has yet been shown to increase the contractile output of an engineered muscle above that of a conventional passive scaffold.

Beyond promoting tissue formation, future materials should also provide long-term mechanical compatibility with dynamic robotic environments [77,78,79,101]. Self-healing and remodeling hydrogels, based on reversible crosslinking mechanisms, represent promising strategies for extending actuator lifetime by allowing materials to recover after repeated mechanical loading [77,78,79,101]. Similarly, computational models capable of predicting material behavior, cellular organization, and tissue remodeling may enable the rational optimization of biofabricated constructs prior to experimental implementation [105,110].

Scaling biohybrid actuators from millimeter-scale demonstrations to centimeter-scale functional systems remains a major challenge and will require fabrication approaches that preserve biological organization across multiple length scales [100,111]. Native muscle achieves functionality through hierarchical organization, where sarcomeric contractile units assemble into aligned fibers and larger tissue structures [100]. Replicating this organization requires fabrication methods to maintain microscale cellular alignment while providing sufficient structural support and transport pathways at larger scales [100,111]. Future manufacturing strategies will therefore likely combine high-resolution fabrication with engineered transport networks, modular assembly, and multiscale scaffold architectures [72,73,74,75,108,112].

Engineered transport strategies, including perfusable networks and microchannel-based designs, will remain important for supporting larger biological constructs by improving nutrient delivery and metabolic support beyond diffusion-limited scales [72,73,74,75]. Current approaches increasingly combine bottom-up cellular self-organization with top-down fabrication of larger conduits, enabling improved metabolic support and integration of engineered tissues [72,73,74,75]. In parallel, fiber-reinforced composites and tunable crosslinking strategies provide opportunities to introduce anisotropic mechanical properties while maintaining the compliance required for cellular function [99,102,109,113,114]. Computational modeling of transport, mechanical stress, and tissue remodeling will further support the rational design of these complex constructs [115,116].

An additional promising strategy for scalable manufacturing is modular biofabrication, in which pre-organized microscale building blocks are assembled into larger functional tissues [108,112,117]. Microgels and engineered tissue modules can be fabricated with controlled cellular organization and subsequently combined to generate larger constructs while preserving local tissue functionality [108,112,117]. Such approaches may provide a practical pathway for overcoming the challenges associated with directly printing large, highly organized biological structures [108,112,117].

Ultimately, manufacturable biohybrid actuators will require not only advanced fabrication technologies but also standardized materials, reproducible cell sources, and optimized production workflows [64,71]. The combination of smart biomaterials, multimaterial printing, modular assembly, and scalable biofabrication strategies may enable the production of reliable actuator platforms suitable for future robotic applications [63,71].

### 6.3. Autonomous Actuators: Adaptive Control, Bioelectronics, and Self-Regulation

The ultimate goal of biohybrid robotics is the development of autonomous actuator systems capable of sensing their environment, regulating their own activity, and maintaining functional performance over extended periods [6,64]. Achieving this capability requires the integration of biological tissues with artificial sensing, stimulation, and control systems that can compensate for the inherent variability and dynamic behavior of living components [4,6].

A major challenge toward autonomous operation is the development of reliable bidirectional communication between biological tissues and engineered electronics [6,64]. Recent advances in flexible electronics, conductive polymers, nanomaterials, and soft robotic substrates have enabled mechanically compatible interfaces that bridge the mismatch between compliant biological tissues and conventional electronic components [79,118,119]. These approaches provide opportunities for integrating stimulation, sensing, and control components directly with muscle-based actuators while maintaining mechanical compatibility with living systems [6,79,118]. Advanced stimulation systems, including charge-balanced biphasic electrical stimulation, may further improve long-term actuation by reducing undesirable electrode–tissue interactions [4,120,121].

An emerging direction is to extend this interface beyond direct muscle stimulation by incorporating neural components into the actuation pathway. Most skeletal muscle biohybrid actuators rely on electrical or optical stimulation applied directly to the muscle, bypassing the neuromuscular interface that physiologically governs skeletal muscle activation [27,122]. Engineering motor neuron–muscle interactions and functional neuromuscular junctions could address this limitation at two levels: supporting aspects of muscle maturation and providing a biologically mediated route for controlling contraction. Rather than acting only as a trigger, the neural component could serve as an intermediate processing layer between artificial control systems and muscle-generated mechanical output, translating externally delivered signals into coordinated neuromuscular responses [123,124]. This architecture could ultimately shift skeletal muscle biohybrid robots from direct external triggering toward neuromuscular control, while creating opportunities to exploit the intrinsic information-processing capabilities of neuronal networks. Achieving this transition, however, will require reproducible innervation, spatial control over neuromuscular junction formation, coordinated neural–muscle maturation, and reliable interfaces through which artificial systems can both modulate and interrogate neural activity.

Beyond stimulation, future biohybrid actuators will require integrated sensing capabilities to continuously monitor both external environments and internal biological states [6,64]. External sensing may provide information about movement, deformation, and interaction with surroundings, while internal sensing approaches, including force measurements, impedance monitoring, microscopy, electrophysiology, and biochemical analysis, could provide information about tissue health, maturation, and fatigue state [6,81]. Combining these measurements with machine learning algorithms may enable real-time estimation of actuator performance and biological condition, allowing a transition from open-loop stimulation toward adaptive, state-aware control [81,119,125,126]. In neuromuscular architectures, such feedback could potentially be directed not only to an artificial controller but also to the neural component itself, creating a route toward systems in which sensing, biological processing, and actuation become functionally coupled.

Adaptive control strategies will therefore become essential for compensating for the nonlinear and time-dependent nature of living actuators [4,6]. Unlike conventional robotic components, engineered tissues continuously change during maturation, training, fatigue, and remodeling, so fixed stimulation protocols may not yield stable performance over time [4,6]. Reinforcement learning, adaptive control, and predictive control strategies could enable biohybrid robots to modify stimulation parameters in response to real-time feedback, maintain stable movement, and compensate for changes in actuator performance [6,127,128]. Future integration of biological tissues with intelligent control architectures may ultimately enable systems capable of autonomous adaptation rather than simple externally triggered movement [6,64].

Integration with soft robotics is expected to extend the functional range of biohybrid actuators, since compliant architectures tolerate the large, low-force deformations that living tissue produces [6,120,129]. Within this space it is worth separating what has been demonstrated from what remains proposed. Integrated electronics and sensing have been shown in living devices: battery-free wireless optoelectronics have driven a walking muscle-powered robot without a tether [21]; embedded gold microelectrodes have provided spatially localised electrical control within a cast cardiac construct [25]; a piezoresistive hydrogel sensor has been co-printed with cells, retaining 87% viability and preserving sensor–tissue interface integrity over ten days of culture [53]; and sensor-embedded muscle has been used to close the control loop in a proprioceptive biohybrid robot [130]. Passive mechanical elements offer an alternative route to the same information, decoding contraction from flexure deflection without a sensor that constrains the tissue [26].

Other combinations remain proposals rather than results. Pairing living muscle with pneumatic elements or wearable interfaces has been suggested as a way to extend the load range and operating environments accessible to biohybrid systems [131,132], but to our knowledge no such hybrid device has yet been demonstrated with living muscle providing the actuation. Identifying that gap is more useful than asserting the capability: it marks a class of device that the compliance argument predicts should work and that no group has yet built.

Future autonomous biohybrid systems may also benefit from materials and architectures capable of self-repair and adaptation [77,78,79,101]. Self-healing materials, dynamic remodeling strategies, and biologically responsive interfaces could help maintain functionality despite mechanical damage or changes in tissue performance [77,78,79,101]. When combined with intelligent sensing and control, these approaches may allow biohybrid robots to operate in environments where continuous human intervention is impractical.

Together, these developments could enable applications ranging from soft robotic systems and adaptive prosthetics to medical devices and advanced in vitro platforms. In medical robotics, biohybrid actuators may provide compliant and responsive actuation strategies for minimally invasive systems and therapeutic technologies [129,132,133]. In prosthetic and assistive technologies, integration with soft sensors and flexible electronics could contribute to more adaptive devices capable of improved interaction with users [119,129,132,133]. Furthermore, engineered biohybrid tissues may provide advanced platforms for drug screening and disease modeling by reproducing physiologically relevant tissue organization and functional responses [134,135,136]. However, successful translation of these systems will ultimately depend on achieving reproducible manufacturing, long-term stability, and reliable integration between biological and engineered components.

## 7. Conclusions

Muscle-based biohybrid actuators have progressed from simple tissue–substrate assemblies to systems capable of coordinated motion and mechanical work. Advances in biofabrication, biomaterials, and stimulation offer increasing control over tissue architecture and integration. However, architectural complexity alone does not ensure reproducible force generation, reliable motion, or sustained operation.

Progress toward robust biohybrid actuators depends on linking tissue development to device performance. Variable maturation limits the reproducibility of contractile output, while tissue–material interfaces determine how effectively that output reaches a load. Neuromuscular interfaces support activation and control; muscle–tendon interfaces support mechanical force transmission. As construct dimensions increase, oxygen and nutrient delivery must also scale to preserve viable, contractile tissue throughout the volume.

Future designs should integrate these biological and mechanical requirements with feedback control and, where useful, wireless stimulation. Wireless operation alone does not establish autonomy: sustained function also requires appropriate metabolic support, reliable sensing, and adaptation to fatigue and tissue remodeling. Shared reporting standards for force, geometry, stimulation, and lifetime would make progress easier to evaluate across laboratories and provide the data needed for predictive design. Bioprinting can contribute by controlling architecture and material placement, but its value should be demonstrated through reproducible functional performance rather than construct size alone.

Rather than projecting clinical application or full autonomy, near-term progress is better judged against four quantities that can be measured today and are seldom reported. The first is *reproducible batch force*: the spread in tetanic force across constructs made in a single batch. Absolute values are available—1.7 mN per muscle ring, and contractile stresses of 0.72 to 4.6 kPa in the best-characterised systems [17,19]—but the variance around them is not. The second is *transmitted-load efficiency*: the fraction of generated contractile force that actually reaches the external load. This separates tissue quality from interface quality, and the gap is already visible, since engineered muscle–tendon junctions withstand more than 5 mN while the tissues driving them generate under 1 mN [20]. The third is *functional lifetime under load*: cyclic operation beyond the two to three weeks currently achieved with antifibrinolytic supplementation [21]. The fourth is *fabrication throughput*: constructs produced per batch and the proportion meeting a stated force specification—the metric on which automated fabrication must eventually justify itself against manual assembly. Reporting these four quantities would let the field be assessed based on evidence rather than based on demonstrations.

## Data Availability

No new data were created or analyzed in this study. Data sharing is not applicable to this article.
